# Temporal Coordination of Carbohydrate Metabolism during Mosquito Reproduction

**DOI:** 10.1371/journal.pgen.1005309

**Published:** 2015-07-09

**Authors:** Yuan Hou, Xue-Li Wang, Tusar T. Saha, Sourav Roy, Bo Zhao, Alexander S. Raikhel, Zhen Zou

**Affiliations:** 1 State Key Laboratory of Integrated Management of Pest Insects and Rodents, Institute of Zoology, Chinese Academy of Sciences, Beijing, China; 2 University of Chinese Academy of Sciences, Beijing, China; 3 Department of Entomology and Institute for Integrative Genome Biology, University of California, Riverside, Riverside, California, United States of America; Virginia Commonwealth University School of Medicine, UNITED STATES

## Abstract

Hematophagous mosquitoes serve as vectors of multiple devastating human diseases, and many unique physiological features contribute to the incredible evolutionary success of these insects. These functions place high-energy demands on a reproducing female mosquito, and carbohydrate metabolism (CM) must be synchronized with these needs. Functional analysis of metabolic gene profiling showed that major CM pathways, including glycolysis, glycogen and sugar metabolism, and citrate cycle, are dramatically repressed at post eclosion (PE) stage in mosquito fat body followed by a sharply increase at post-blood meal (PBM) stage, which were also verified by Real-time RT-PCR. Consistent to the change of transcript and protein level of CM genes, the level of glycogen, glucose and trehalose and other secondary metabolites are also periodically accumulated and degraded during the reproductive cycle respectively. Levels of triacylglycerols (TAG), which represent another important energy storage form in the mosquito fat body, followed a similar tendency. On the other hand, ATP, which is generated by catabolism of these secondary metabolites, showed an opposite trend. Additionally, we used RNA interference studies for the juvenile hormone and ecdysone receptors, Met and EcR, coupled with transcriptomics and metabolomics analyses to show that these hormone receptors function as major regulatory switches coordinating CM with the differing energy requirements of the female mosquito throughout its reproductive cycle. Our study demonstrates how, by metabolic reprogramming, a multicellular organism adapts to drastic and rapid functional changes.

## Author Summary

Mosquitoes transmit numerous devastating human diseases due to their obligatory hematophagy that is required for the efficient reproduction. Metabolism must be synchronized with high energetic needs of a female mosquito for host seeking, blood feeding and rapid egg development. Each reproductive cycle is divided into two phases that are sequentially governed by juvenile hormone (JH) and 20-hydroxyecdysone. During the pre-blood meal phase, the JH receptor Methoprene-tolerant (Met) controls carbohydrate metabolism (CM) pathways and its RNA interference (RNAi) silencing caused up-regulation of CM enzymes at the transcript and protein levels activating glycolytic flux and depletion of storage and circulating sugars. During the second, post blood meal phase, CM was regulated by the ecdysone receptor EcR and its RNAi silencing had a dramatic effect opposite to that of Met RNAi. Thus, we show that Met and EcR function as regulatory switches coordinating carbohydrate metabolism with energetic requirements of the female mosquito reproductive cycle.

## Introduction

The ability of multicellular organisms to maintain metabolic homeostasis and respond to changing energy requirements during development, reproduction and stress represents an essential adaptation critical for survival and evolutionary success. Thus, it is important to decipher regulatory mechanisms coordinating metabolic pathways; understanding these mechanisms in organisms facing extreme and fluctuating energy demands is particularly valuable.

Female mosquitoes, which are obligatory blood feeders, serve as disease vectors [1]. Pathogens, taking advantage of this blood dependency, use mosquitoes as vectors spreading serious human diseases. Despite continuing efforts and advances in insect control, mosquitoes pose an enormous threat, killing over a million people each year. The situation is aggravated by the lack of effective vaccines, fast growing insecticide resistance, social complexities and ecological changes [2]. A detailed understanding of the reproductive biology of the mosquito may provide vital information to take us a step closer to more effective vector-control strategies.

Hematophagous mosquitoes possess numerous distinct physiological features that play a critical role in the stunning environmental adaptations of these disease vector insects. These include a powerful system of odorant receptors, an extremely efficient host-seeking behavior, adaptations for blood feeding and digestion, ability to excrete large amounts of solutes, and rapid egg development [3]. Hematophagy puts extremely high energy demands on a female mosquito at different stages throughout its reproductive cycle. Therefore, metabolic pathways must be synchronized with energy needs of a reproducing female mosquito. However, regulatory mechanisms governing temporal coordination of metabolism at the molecular level have not been well understood in mosquitoes.

Each reproductive cycle of a female mosquito is divided into two phases, which are governed by alternating titers of two major insect hormones—a sesquiterpenoid juvenile hormone (JH) and a steroid hormone 20-hydroxyecdysone (20E). JH guides the female mosquito development from the adult eclosion from pupae to blood feeding. During this JH-controlled post eclosion (PE) phase, which lasts 3–5 days, a female mosquito matures and prepares itself for events associated with subsequent blood feeding, while actively seeking hosts.

Ingestion of blood leads to dramatic events in a female mosquito, including digestion of a huge meal, powerful excretion, a high level of gene expression and rapid egg maturation. During this post blood meal (PBM) phase, a female mosquito faces an intense degree of metabolic activity. 20E is the major regulator of the PBM phase of the female mosquito reproductive cycle, and its action is mediated by a nuclear receptor, the Ecdysone Receptor (EcR) [4]. The mosquito fat body serves as the nutrient sensor organ, detecting the nutrients derived from a blood meal and blood-derived nutrients are utilized for the production of YPPs in fat body cells [1].

In this study, we investigated whether the two major regulators of female mosquito reproductive cycles, JH and 20E, are involved in the temporal coordination of CM throughout the female mosquito reproductive cycle. Our results show that the JH receptor, Met, and the EcR synchronize CM with energy requirements of a reproducing female mosquito. Deciphering the regulatory mechanisms governing mosquito CM has shed light on adaptations of an organism dealing with intense energetic stress.

## Results

### Expression dynamics of carbohydrate metabolism genes throughout the female mosquito reproductive cycle

**Fig 1:**
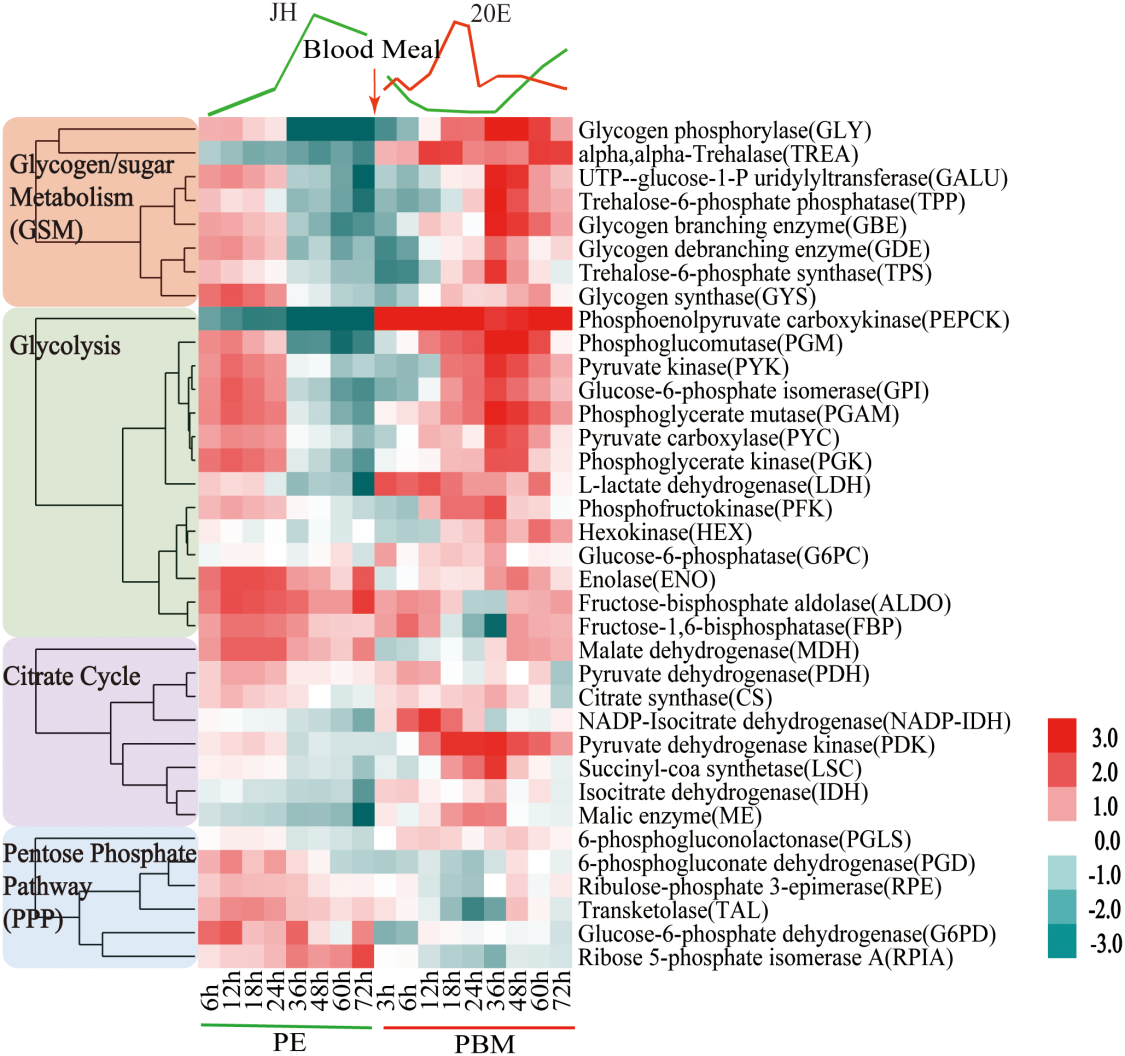
Heatmap representing the microarray-based expression pattern of CM genes. Candidates with a fold change greater than 1.75 (0.8 in log2 scale) and a false-discovery rate (*p* value) less than 0.01 are included. Gene expression time points in the PE and PBM phases were normalized to 6h PE and 72h PE time points, respectively. The four shades of color to the left represent different CM pathways: red, glycogen/sugar metabolism; green, glycolysis; purple, citrate cycle; and blue, pentose phosphate pathway. The respective dendograms generated by hierarchical clustering of genes from each pathway are also provided.

We characterized transcript abundance of genes encoding CM enzymes in the fat body of the female *Aedes aegypti* mosquito throughout the first reproductive cycle. For this analysis, we used two time course fat body microarray transcriptomes spanning the entire first gonadotrophic cycle; one that encompassed eight time points from 6h to 72h PE, and a second one covering nine time points from 3h to 72h PBM (S1 and S2 Tables). Both transcriptomes were obtained from custom-made Agilent microarray chips that contained probe sets corresponding to 15,321 genes in the *A*. *aegypti* genome [5]. DEG sets during PBM were calculated by comparing transcripts from each of the nine time points with that at 72h PE using the same filtering criteria as those for the PE genes [5]. CM gene transcripts were abundant during the first 24h PE, while later, by 72h PE, there was a considerable decline in their levels (Fig 1 and S1A Fig). Following a blood meal, most CM genes exhibited significant up-regulation reaching their maximal expression by 36h PBM, dropping back to early PE levels by 72h PBM (Fig 1 and S1A Fig). Overall, the second wave of the CM gene activity during the PBM phase was considerably higher than the first during the PE phase. The genes encoding glycogen/sugar metabolism (10 of 17 enzyme coding genes) and glycolysis (13 of 28 enzyme coding genes) exhibited particularly pronounced fluctuations in their expression levels (Fig 1 and S1B and S1C Fig). The genes encoding the citrate cycle exhibited a similar trend, but to a lesser extent (Fig 1).

**Fig 2:**
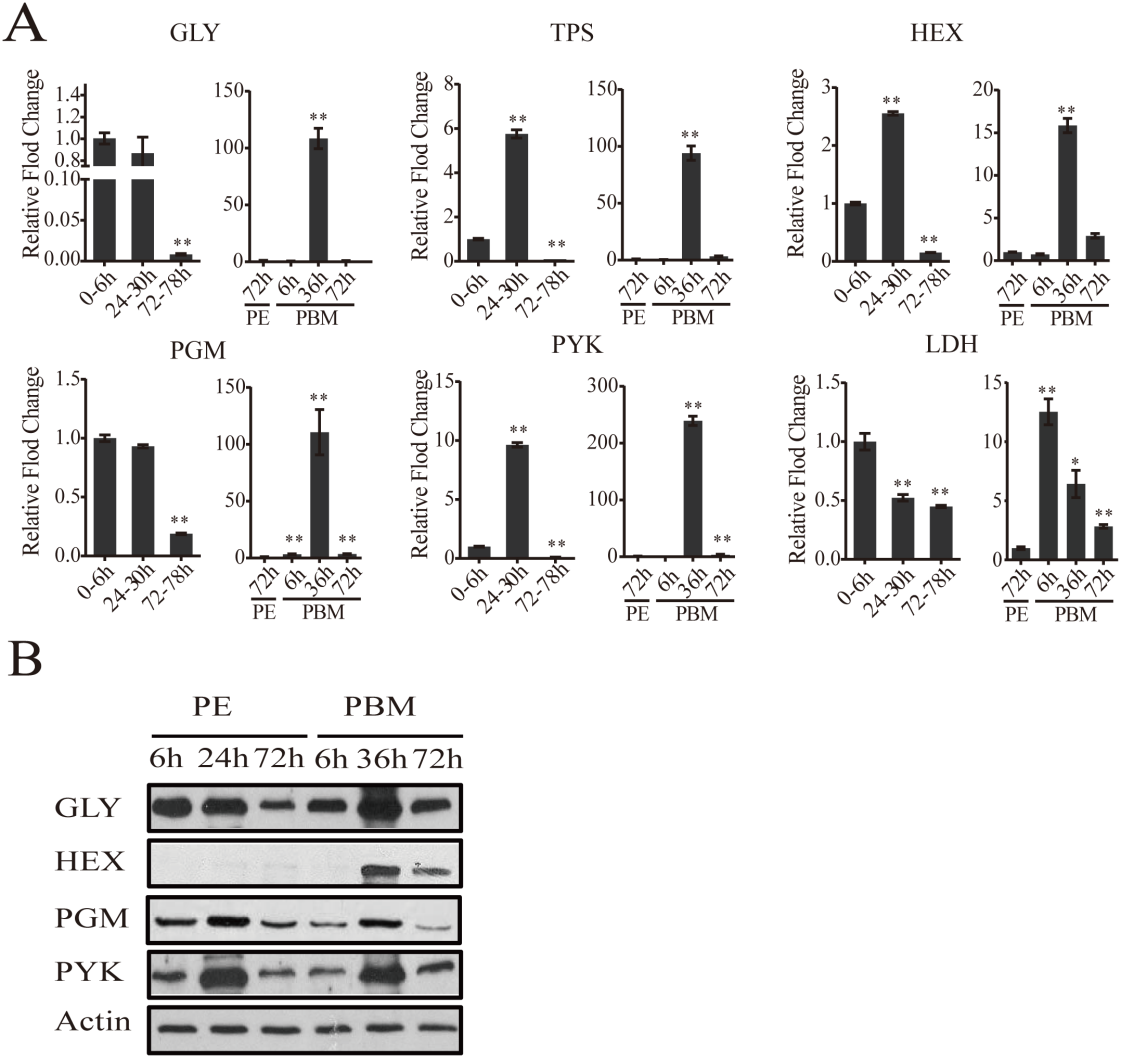
CM gene expression dynamics in the fat body of adult female mosquitoes. (A) qPCR analysis of selected CM genes during PE and PBM. Relative abundance of PE and PBM time points were normalized to the 0-6h PE and 72h PE, respectively. In the graphs, the abundance of these two time points is represented as 1.0, with corresponding adjustments for other time points. All experiments were performed in triplicate, with similar results. Error bars represent ± SD. * *p* < 0.05;** *p* < 0.01. Additional CM genes are shown in S2 Fig (B) Western blot showing the fat body protein levels of key CM enzymes during PE and PBM. Total protein extracts were prepared from eight adult female fat bodies for each indicated time point. Actin was used as a control for loading and transfer.

To authenticate our microarray analysis results, transcript levels of genes encoding key enzymes of CM pathways in fat body samples were determined using real-time PCR (qPCR) (Fig 2A and S2 Fig). In agreement with microarray data, the transcript of the glycogen phosphorylase gene (*GLY*) encoding the key glycogen degrading enzyme was high at the beginning of the PE phase, but its expression at 72h PE was dramatically reduced (Fig 2A). This is in contrast to a small decrease in the mRNA levels of genes encoding the enzymes involved in glycogen biosynthesis—glycogen synthase (*GYS*) (S2 Fig). These gene dynamics suggest predominance of glycogen accumulation during the PE phase. During the PBM phase, the *GLY* transcript was greatly elevated at 36h PBM (Fig 2A), while that of the *GYS* gene showed only a moderate increase, suggesting a trend opposite to PE in the utilization of sugar reserves during the PBM stage (S2 Fig). Transcripts of genes encoding enzymes for trehalose metabolism, trehalose-6-phosphate (*TPS*) and trehalose-6-phosphatase (*TPP*) and trehalase (*TREA*) (Fig 2A and S2 Fig), the enzymes responsible for transforming glucose to trehalose, declined during the PE phase. During PBM, each of these three genes had a dramatic peak of expression at 36h PBM (Fig 2A and S2 Fig). Transcript levels of nine glycolytic genes, determined using qPCR, were in agreement with microarray data showing differential PE and PBM expression of these genes (Fig 2A and S2 Fig). Our analysis included three genes encoding the rate-limiting enzymes of glycolysis—hexokinase (*HEX*), phosphofructokinase (*PFK*), pyruvate kinase (*PYK*) (Fig 2A and S2 Fig). Although they followed a similar expression trend during the mosquito reproductive cycle, the levels of their relative expression differ significantly (Fig 1). PYK catalyzes the final glycolytic irreversible step generating pyruvate and ATP [6]. Strikingly, the *PYK* gene expression was highly elevated during the mosquito gonadotrophic cycle, particularly during the PBM stage when its transcript increased over 200-fold, while PFK transcript increased more than 125 folds (Fig 2A). This suggests a dramatic acceleration of the glycolytic flux after blood feeding. Lactate dehydrogenase (LDH) is the enzyme that catalyzes conversion of pyruvate to lactate, and this reaction supplies NAD^+^ [7]. Notably, there was a 12-fold elevation in the *LDH* gene transcript level by 6h PBM, suggesting a drastic increase in the generation of lactate immediately after a blood meal (Fig 2A).

### Alteration of CM enzyme protein levels during the mosquito reproductive cycle

Using antibodies that recognize respective *Aedes* CM enzymes at the protein level (S3 Table), we performed western blot analyses of samples from 6h, 24h, 72h PE and 6h, 36h, 72h PBM developmental time points. Protein levels for the major glycogen-utilizing enzyme, GLY, and two key glycolytic enzymes, PGM and PYK, were high until 24h PE, after which there was a drop, at 72h PE. During the PBM period, all three proteins were in abundance at 36h (Fig 2B). HEX, the enzyme that catalyzes the first step in glycolysis, converting glucose to glucose-1-P, showed a weak accumulation at 72h PE in female mosquito fat bodies. However, this HEX protein could be detected at a much higher level at 36h PBM. Protein levels of all four tested enzymes declined during the late PBM phase (72h PBM) (Fig 2B). Overall, our western blots demonstrate that the protein levels of glycolysis and glycogen/sugar metabolic enzymes exhibit periodic changes throughout the mosquito reproductive cycle that correlate with the transcript abundance of their respective genes.

### Levels of storage and circulating sugars and triacylglycerols (TAG) during the female mosquito gonadotrophic cycle

To find out whether sugar reserves correlated with fluctuating levels of CM enzymes at the gene and protein levels in the female mosquito fat body, we undertook thorough quantitative measurements of stored and circulating sugars during the female mosquito reproductive cycle.

**Fig 3:**
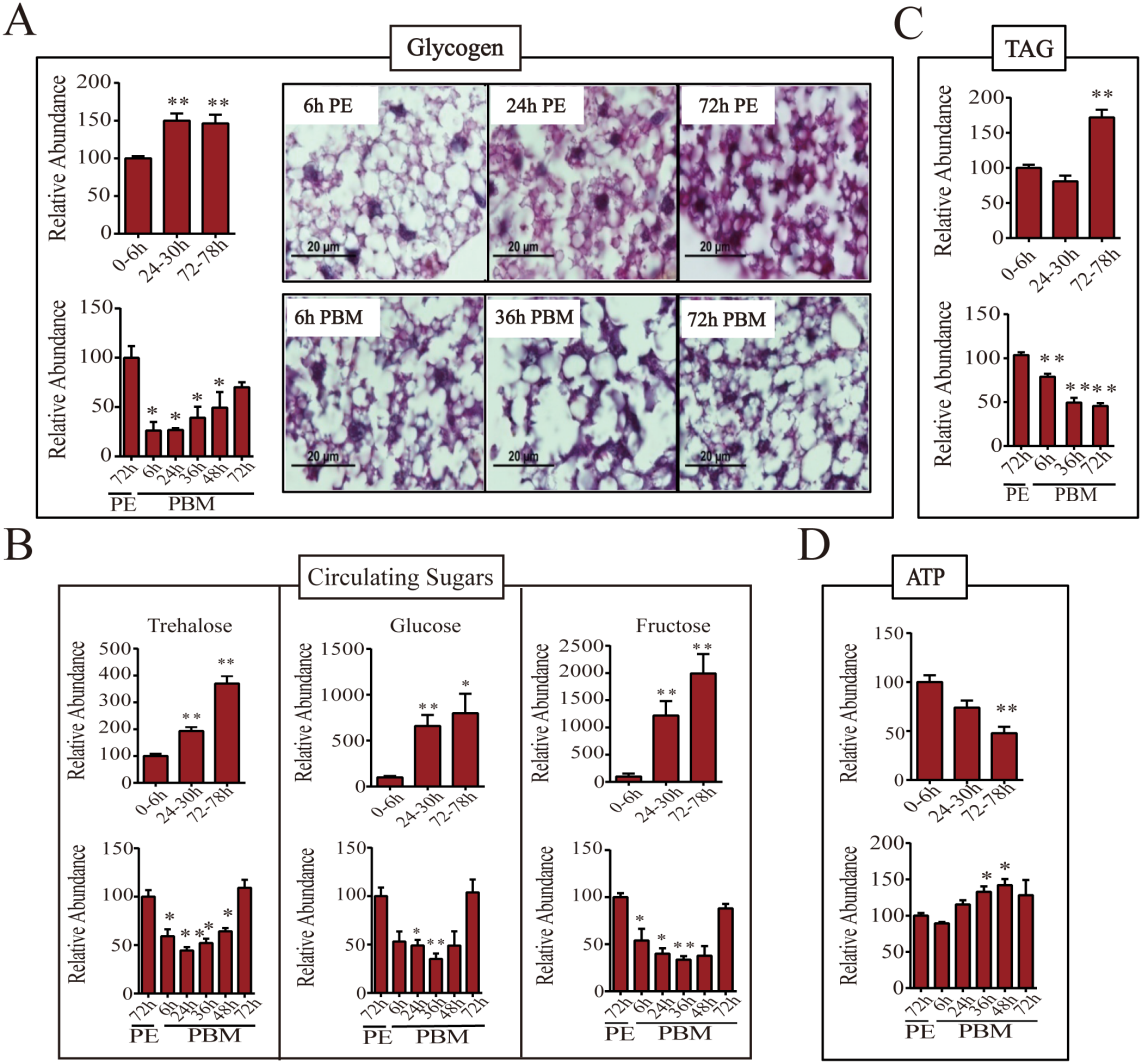
Levels of storage and circulating sugars, TAG and ATP during PE and PBM phases. (A) Endogenous levels of glycogen during PE (*top panel*) and PBM (*bottom panel*) phases. Total glycogen content of adult female mosquitoes was measured colorimetrically (*left*; n = 6 independently collected samples per time point, with six mosquitoes per sample). Glycogen content of the female mosquito fat body was also visualized by PAS staining (*right*). Similar results were observed in two additional independent experiments. (B) Levels of circulating sugars trehalose (*top*), glucose (*middle*) and fructose (*bottom*) for PE and PBM as determined by means of gas chromatography—mass spectrometry (GC-MS; n = 12 independently collected samples per time point, with six mosquitoes per sample). (C) TAG levels were measured during PE (*top panel*) and PBM (*bottom panel*) phases (n = 6 independently collected samples per time point, with six mosquitoes per sample). (D) ATP concentrations in female mosquitoes during the PE (*top*) and the PBM (*bottom*) phases were measured using high performance liquid chromatography (HPLC; n = 6 independently collected samples per time point, with six mosquitoes per sample). Amounts of glycogen (A, *left*), trehalose, glucose and fructose (B), TAG (C), and ATP (D) were normalized to total protein levels. The metabolite levels at 0-6h PE and 72h PE are represented as 100, with relative adjustments in the sugar and ATP levels at other time points. Error bars represent ± SD. * *p* < 0.05;** *p* < 0.01.

In a newly eclosed female mosquito, the glycogen level was relatively low but increased significantly by 24h PE (about 150% of that of 6h PE) and was maintained at a similar level for the rest of the PE developmental phase (Fig 3A). A blood meal, however, triggers glycogen depletion and by 24h PBM its level dropped to about half that of the late PE phase mosquitoes. The glycogen level increased by 72h PBM, but was still lower than that of 72h PE mosquitoes (Fig 3A). In order to visualize the glycogen content *in situ*, we used Periodic acid/Schiff (PAS) staining of fixed female adult mosquito fat bodies. Glycogen content was at a detectable level at 6h PE. Consistent with our colorimetric measurements of glycogen, there was a significant increase in PAS positive signal in the 24h PE fat body. Using this staining method, however, the highest glycogen level was observed at 72h PE (Fig 3A). Correlating well with glycogen level measurements, PAS staining showed that glycogen content was less at 6h PBM than at 72h PE. Staining also revealed that the glycogen levels were moderately increased from 6h to 72h PBM in the fat body (Fig 3A).

We then measured the levels of circulating sugars using gas chromatography—mass spectrometry (GC-MS). Trehalose level increased during the PE phase, reaching its maximum at 72-78h PE (about 3-fold increase in comparison with that at 0-6h PE) (Fig 3B). A blood meal triggers depletion of trehalose and by 24h PBM its concentration dropped to about half that of late-stage PE mosquitoes. During late PBM, the level of trehalose increased and returned to original level by 72h PBM (Fig 3B). Although trehalose is the major form of the circulating sugar in insects, glucose and fructose function as additional circulating sugars found in the hemolymph [7,8]. During the late PE phase, there was approximately 10- and 20-fold increase in glucose and fructose levels, respectively (Fig 3B). Blood feeding resulted in a decrease in the levels of these two sugars until 36h PBM, after which it was restored back to PE levels by 72h PBM (Fig 3B).

Triacylglycerols (TAG) represent another important energy storage form in the mosquito fat body. During the PE phase, the change in TAG level was delayed compared with that of glycogen. The TAG level was relatively low from 6 to 24h PE, but increased by 72h PE (Fig 3C). During PBM phase, the TAG levels in the fat body dropped significantly at 6h PBM reaching its lowest level by 72h PBM (Fig 2C).

Adenosine triphosphate (ATP) serves as a major indicator of energy consumption by an organism [7]. To evaluate energy utilization in female mosquitoes throughout the reproductive cycle, we measured ATP levels using high performance liquid chromatography (HPLC). The ATP level was high in newly eclosed female mosquitoes at 6h PE, declining thereafter, and by 72-78h PE its level was only 50% of that of 6h-old mosquitoes (Fig 3D). However, the ATP level increased during the PBM phase, reaching a peak at 48h PBM, which was higher than that at 72h PE (Fig 3D).

### Changes in intermediary metabolites during the female mosquito gonadotrophic cycle

**Fig 4:**
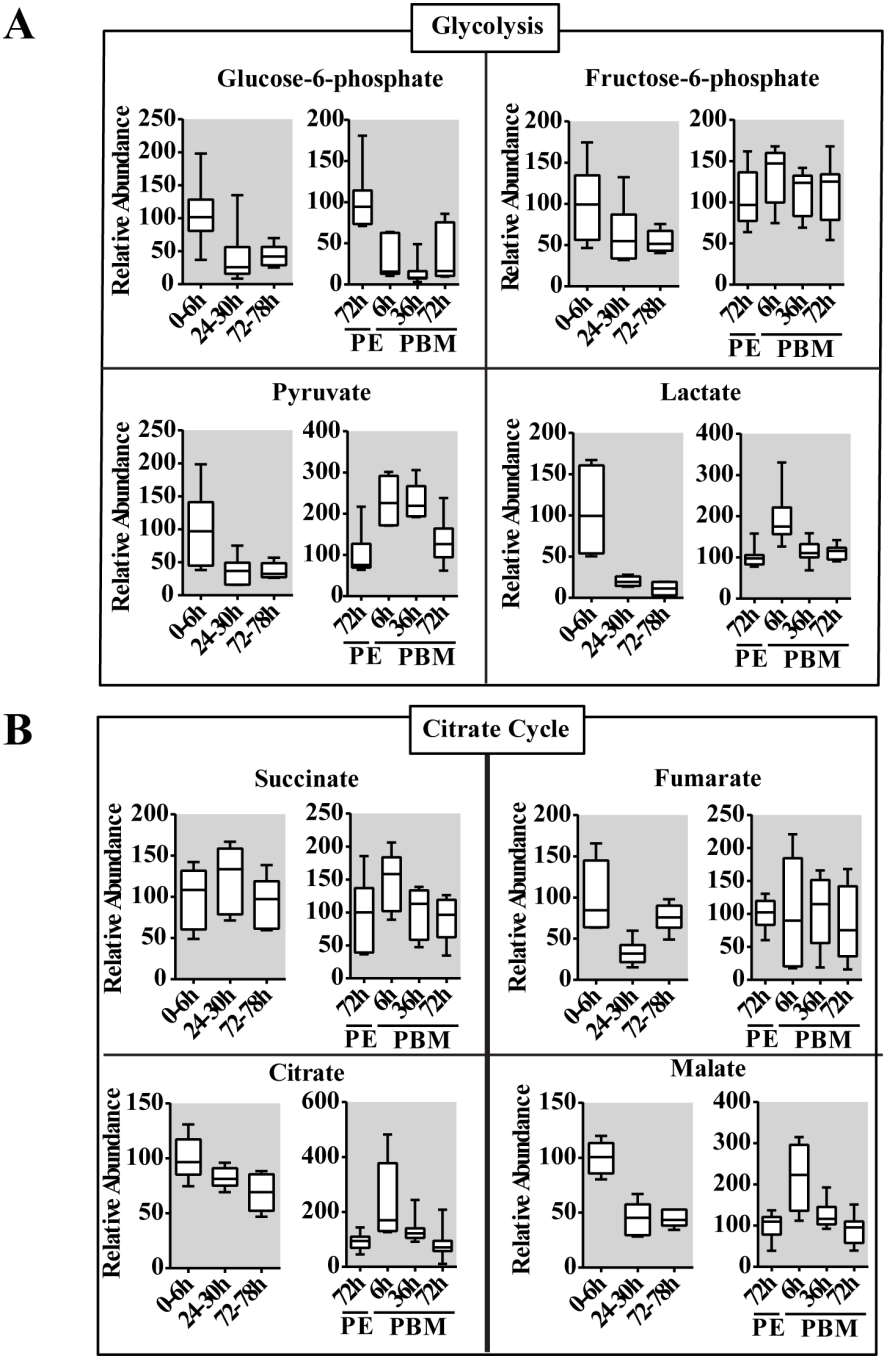
Changes in the level of intermediary metabolites throughout the reproductive cycle of adult female mosquito. (A and B) Relative levels for intermediary metabolites of glycolysis (A) and the citrate cycle (B) during PE and PBM development. Glucose-6-phosphate, fructose-6-phosphate, pyruvate and lactate from the glycolytic pathway, and citrate, succinate fumarate and malate from the citrate cycle were measured using gas chromatography—mass spectrometry (GC/MS). The time points 0-6h PE and 72h PE were used as controls for PE and PBM periods, respectively. The controls are represented as relative abundance 100, and the levels at other time points were adjusted accordingly. In the box plots, the box represents the lower and upper quartiles, the horizontal line represents the median and the bars represent the minimum and maximum data points (n = 12 samples collected from independent populations, with six adult female mosquito per sample). Three independent sets of metabolite measurements were performed with similar results.

To provide further insight into the CM dynamics in reproducing female mosquitoes, we used GC-MS to measure several intermediary metabolites (IMs) of glycolysis and the citrate cycle. Overall, this analysis revealed that IM profiles correlated with those of CM enzymes, exhibiting two pronounced waves at the PE and PBM phases, respectively (Fig 4). Glucose-6-phosphate represents the first key IM of the glycolytic pathway that also serves as a precursor for glycogen/sugar metabolism and pentose-phosphate pathways. During the PE phase, the level of glucose-6-phosphate was reduced 2-fold by 72-78h (Fig 4A). This metabolite also showed a significant drop in its level immediately after a blood meal, at 6h PBM, and its level remained low throughout the PBM phase, being elevated only by 72h PBM (Fig 4A). The level of the next glycolytic IM, fructose-6-phosphate, exhibited a 2-fold reduction by 72–78 h PE, but it was elevated at 6h PBM, maintaining its high level throughout the rest of the PBM phase (Fig 4A). There was a dramatic reduction in the level of pyruvate, the terminal product of glycolysis during the PE phase. However, more than a 100% increase of the pyruvate level was observed at 6h PBM, reflecting an increase in the glycolytic flux following the blood intake. The pyruvate level remained high until 36h PBM, declining thereafter (Fig 4A). Our transcript data analyses demonstrated a reduction in the mRNA level of the gene encoding LDH, the enzyme catalyzing transformation of pyruvate to lactate, at the end of PE period (Fig 1). Accordingly, GC-MS measurements of lactate showed a pronounced drop in its level late PE. Moreover, there was an elevation in the lactate level during the PBM phase corresponding to the rise in the expression of this gene (Fig 4A).

The citrate cycle is the key pathway used for energy production in all aerobic organisms. Pyruvate serves as an essential precursor for the citrate cycle, and its availability along with activity levels of citrate cycle enzymes determines the final outcome. The latter can be determined by measuring concentrations of citrate cycle IMs. The GC-MS analysis revealed considerable differences in PE and PBM profiles of citrate cycle IMs, which reflects contrasting energetic requirements of the female mosquito during these two phases of the reproductive cycle (Fig 4B). The level of citrate, the first IM of the citrate cycle, exhibited a significant reduction over the PE phase, while it was highly elevated at 6h PBM. Succinate and fumarate exhibited more moderate fluctuations. Malate, however, had PE and PBM profiles similar to those of citrate (Fig 4B).

### The JH receptor, Met, functions as a regulatory switch of CM during the PE phase

To investigate the role of JH in regulation of CM during PE phase, we topically applied JH III onto newly eclosed female mosquitoes and investigated the effect of this treatment 20h later. The application of JHIII caused a premature drop in abundance of CM gene transcripts (S3A Fig). At the same time, there was a significant elevation in the levels of glycogen and glucose compared with control untreated mosquitoes (S3B Fig).

**Fig 5:**
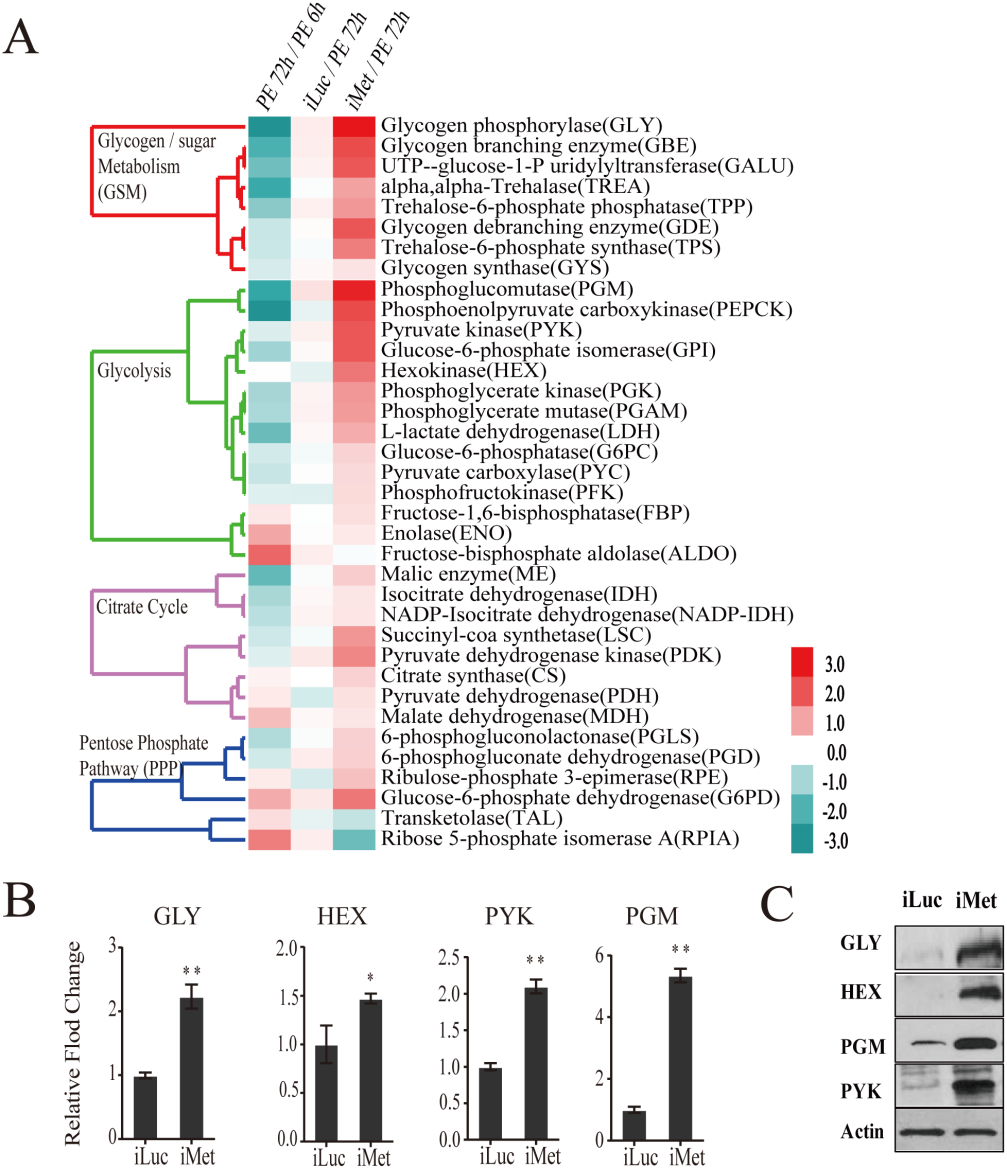
CM genes are upregulated in the Met-depleted mosquito. (A) Heatmap showing the alteration of CM gene expression in the Met-depleted mosquito (iMet) compared with that in the control samples iLuc and 72h PE, as characterized by microarray analysis. The raw intensity values for expression of CM genes in iMet and iLuc samples were normalized to 72h PE, while the 72h PE values were normalized to 6h PE samples. Only genes showing a fold change > 1.75 (0.8 in log2 scale) with a false-discovery rate (*p* value) less than 0.01 were included. Hierarchical clustering dendograms for the four different CM pathways are provided. Dendograms are color coded for glycogen/sugar metabolism (red), glycolysis (green), citrate cycle (purple) and pentose phosphate pathway (blue). (B) qPCR validations for a selected set of genes from glycogen/sugar metabolism and glycolysis, showing the effect of Met depletion on these genes at the transcript level. Relative abundance of control iLuc is represented as 1, with corresponding adjustments in iMet value. Error bars represent ± SD. * *p* < 0.05;** *p* < 0.01. (C) Western blots showing dramatic changes in CM enzymes at the protein level in the fat body of the Met-depleted mosquitoes during PE development. Actin was used as a loading control.

Our previous data indicated that the JH receptor Met plays a central role in regulating JH-mediated gene expression in the fat body of the PE female mosquito [5]. Met silencing has been shown to inhibit ovarian follicle growth as well as result in the reduction of the egg number [5,9]. We examined the transcriptome obtained from the fat body of Met RNAi-depleted females and analyzed the response of CM genes. The CM gene transcripts were enriched among upregulated gene cohorts of the iMet transcriptome (S4A Fig). The transcripts of genes belonging to glycogen/sugar metabolism and glycolysis were particularly upregulated, while those of the citrate cycle were elevated to a significantly lesser degree (Fig 5A and S4 Table). Next, we silenced Met by RNA interference (RNAi) in female mosquitoes (iMet) at 24h PE and analyzed transcript levels of CM genes 4 days later using qPCR. We measured mRNA levels of four glycogen/sugar metabolism genes—*GLY*, *TPS*, *TPP*, and *TREA*—in the Met-depleted background and found these genes to be considerably induced (Fig 5B and S4C Fig). Six glycolytic enzyme coding genes, including the rate limiting *PYK* and *HEX*, were significantly upregulated in the iMet mosquito fat body (Fig 5B and S4C Fig). We also tested the key rate-limiting enzyme PFK but found no effect of Met, consistent with microarray results (S4C Fig). Western blot analysis showed a substantial accumulation of enzymes for glycogen/sugar metabolism and glycolysis at the protein level in fat bodies of Met-silenced female mosquitoes (Fig 5C). These results demonstrate a dramatic effect of Met RNAi knockdown on CM gene and protein levels, suggesting that the JH receptor plays a critical role in CM regulation.

**Fig 6:**
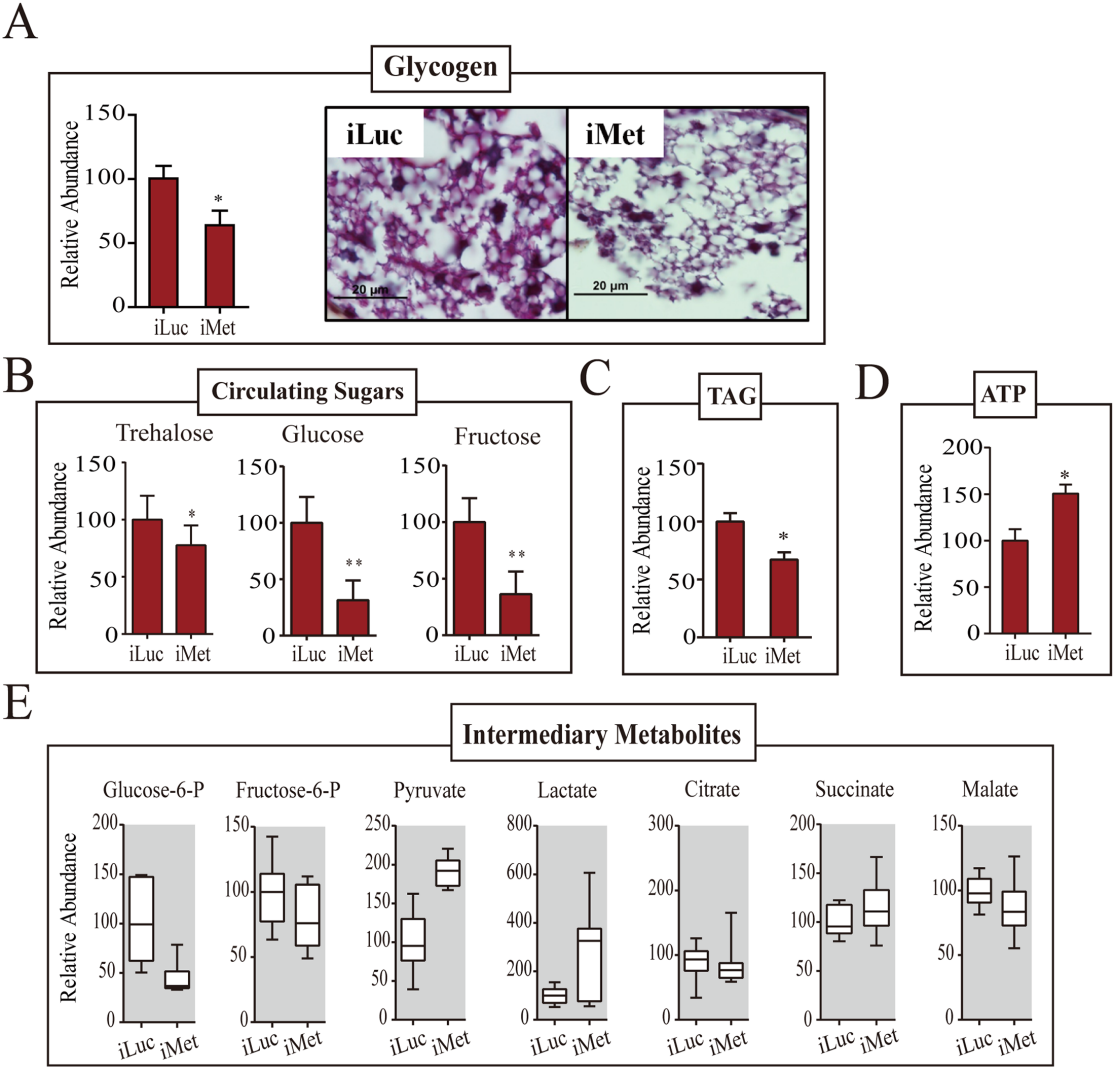
Met-depleted mosquitoes exhibit metabolic defects. (A) A decrease in the glycogen levels was observed in Met-depleted adult female mosquitoes, as measured by colorimetry (*left*) and PAS staining (*right*). Mosquitoes injected with iLuc were used as controls. (B) Changes in the levels of circulating sugars as a result of Met depletion. A reduction in trehalose, glucose and fructose concentrations were observed in iMet mosquitoes in comparison with the iLuc controls. (C) A decrease in the TAG level was observed in Met-depleted adult female mosquitoes, as measured by colorimetry. (D) ATP levels in iMet mosquitoes as determined by HPLC. A significant increase in the ATP levels was observed in iMet mosquitoes when compared with controls. Experimental procedure, normalizations and statistical analysis are similar to those in Fig 2, parts A-C, respectively. (E) Box plots showing the relative levels of intermediary metabolites of CM in iMet mosquitoes. While lactate and the glycolytic end product pyruvate were significantly induced, a reduction in the levels of glucose-6-phosphate and fructose-6-phosphate was observed. Intermediates of the citrate cycle, citrate, succinate and malate showed a mild increase in concentration. Three independent batches of experiments were performed, each showing similar results. Tissue collections and samplings were performed 4 days post-injection. All metabolite levels were normalized to the amount of total endogenous proteins. The control iLuc is represented as relative abundance 100, with corresponding adjustments in iMet levels. Error bars represent ± SD. * *p* < 0.05; ** *p* < 0.01.

The glycogen levels were significantly reduced in Met-silenced female mosquitoes (Fig 6A and 6B). A dramatic depletion of glycogen reserves in fat bodies of Met-silenced female mosquitoes was confirmed by means of PAS staining (Fig 6A). Circulating hemolymph sugars—trehalose, fructose and glucose—significantly declined in abundance in Met-depleted female mosquitoes (Fig 6B). Like the sugar reserves, TAG levels also declined after Met depletion (Fig 6C). Met RNAi depletion resulted in elevated ATP levels, showing an increase in energy consumption in these mosquitoes (Fig 6D). We then measured levels of pyruvate and lactate, the metabolic end products of glycolysis, both of which were significantly elevated with Met depletion, indicating that Met affects the glycolytic flux. However, citrate, succinate and malate, IMs of the citrate cycle, showed no noticeable fluctuations in response to Met depletion (Fig 6E). Our data show that CM is severely compromised in Met-silenced female mosquitoes. This effect of Met RNAi silencing clearly demonstrates that Met functions as a major regulatory switch of CM during the PE phase of the gonadotrophic cycle.

### 20E and amino acids are important for regulation of CM during the PBM phase

20E and the Amino Acid (AA)/Target of Rapamycin pathway have been implicated in regulating vitellogenic events in female mosquitoes [10,11,12]. To test whether AAs and 20E affect CM gene expression in the female fat body, we used an *in vitro* tissue culture assay in which fat body tissue isolated from mosquitoes at 72h PE was incubated in the presence of AAs and/or 20E [11,13]. Incubation of the fat body in AA-containing medium elevated transcript abundance of *PYK* and *GLY*, while addition of 20E to this medium resulted in a further rise of their levels (S5A Fig). To investigate whether these regulatory factors were also involved in controlling CM metabolism during the PBM phase, female mosquitoes 72PE were injected with 20E and AAs. The expression of *GLY* and *PYK* were upregulated as a result of the simultaneous application of AAs and 20E; *LDH* was responsive to AAs but not 20E, while *GYS* to neither of these two regulators (S5B Fig). In agreement with in vitro experiments, AAs elevated LDH transcript abundance, but 20E had little effect. Thus, 20E and AAs play different roles in the regulation of CM genes. In in vivo experiments utilizing application of 20E and AAs, glycogen and glucose levels decreased when both AAs and 20E were given, mimicking the status of these sugars in PBM mosquitoes (S5C Fig).

### The EcR is a critical regulator of CM during the PBM reproductive phase

**Fig 7:**
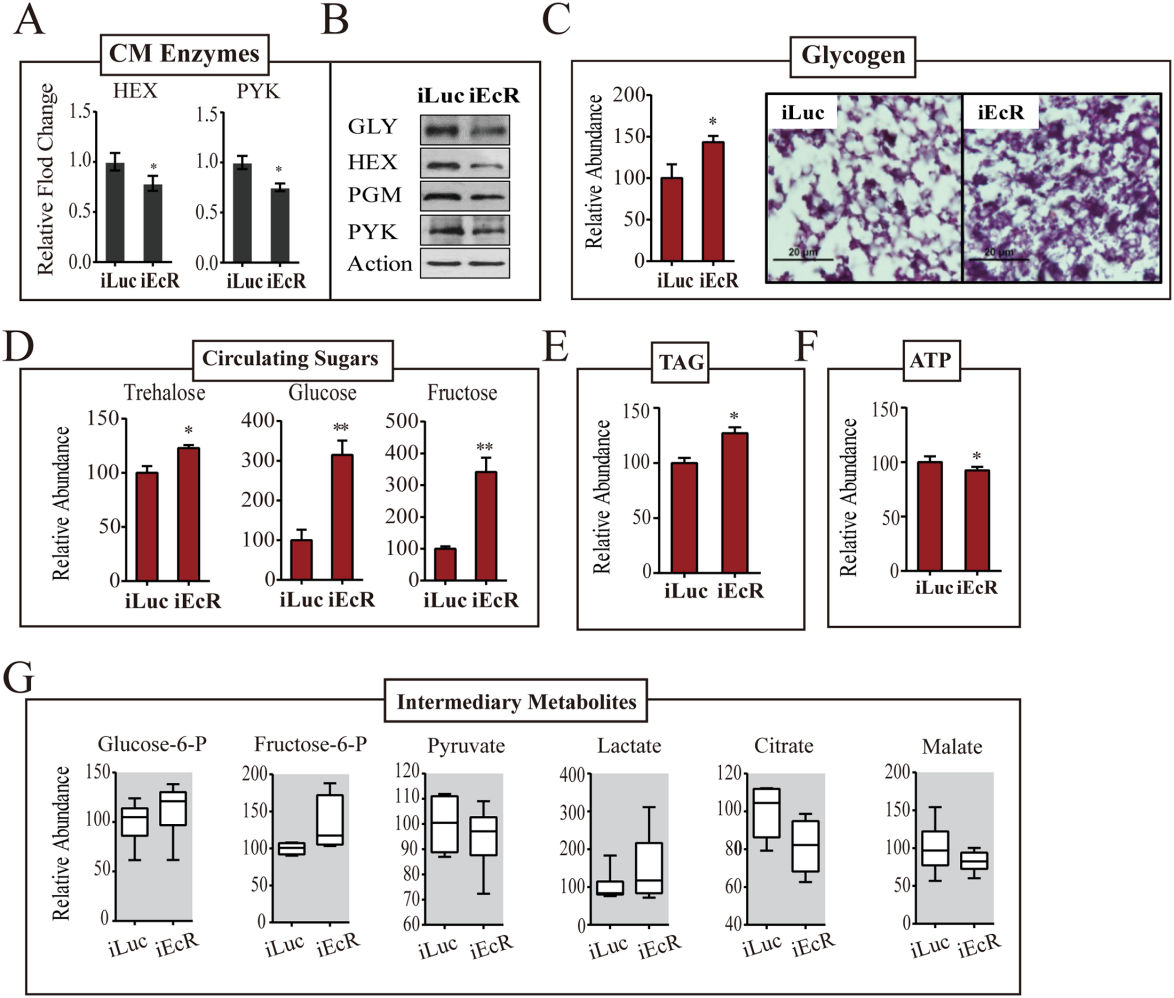
EcR depletion affects CM during PBM development. (A) qPCR-based transcript levels of CM enzymes in iEcR adult female mosquito. Transcripts of HEX, GLY, PGM and PYK were significantly repressed as a result of EcR knockdown.(B) Western blot showing the protein levels of enzymes GLY, HEX, PGM and PYK in EcR-depleted mosquitoes. Actin was used as a loading control. (C) A moderate increase in the glycogen levels was observed as result of RNAi depletion of EcR. Glycogen levels measured by colorimetry (*left*) and PAS staining (*right*). (D) Effect of EcR knockdown on the concentration of circulating sugars. While dramatic increases in the glucose and fructose levels were noted as a result of EcR depletion, trehalose was only mildly altered. Gas chromatography—mass spectrometry (GC-MS) was used for the quantification of circulating sugars. (E) An increase in the TAG levels was observed in Met-depleted female mosquitoes, as measured by colorimetry (F) High-performance liquid chromatography (HPLC)-based ATP measurements in iEcR mosquitoes. A moderate reduction in ATP level was observed in treated mosquitoes. (G) Box plots showing the variation of relative level for small metabolites of the CM pathway in the EcR-depleted mosquitoes. Small metabolites were normalized to the total protein level of the organisms. Measurements from three independent biological experiments were performed, with similar results. Experiments were performed in triplicate using separate cohorts of mosquitoes. For all experiments, iLuc mosquitoes were used as controls. iEcR and iLuc mosquitoes were blood fed 4 days post-injection, followed by sample collections at 36h PBM. The control iLuc is represented as relative abundance 100, with corresponding adjustments in iEcR levels. Error bars represent ± SD. *p < 0.05;**p < 0.01.

20E is the principal hormone governing PBM reproductive events in female mosquitoes. EcR silencing has been reported in mosquitoes with EcR knockdown resulting in reduced ovarian follicular length [14], and egg numbers as compared to the controls (S6A Fig). Therefore, we investigated whether EcR plays a role in controlling CM. We silenced EcR using dsRNA to a common EcR region (iEcR) in female mosquitoes at 24h PE, blood fed them 4 days later, and analyzed transcript levels of CM genes at 36h PBM using qPCR (S6B Fig). Expression of *TREA* and *TPP* genes encoding enzymes of glycogen/sugar metabolism were transcriptionally suppressed at 36h PBM as a result of EcR silencing (S6C Fig). Representative glycolytic genes—*HEX*, *PFK* and *PYK*—were controlled by the EcR in a similar manner (Fig 7A and S6C Fig). *GLY*, *PGM* and *GPI* followed the same trend. In contrast, *GYS* and *LDH* were not affected by EcR RNAi silencing. This is in agreement with the lack of 20E effect on expression of these genes described above. This transcriptional alteration was also reflected in enzyme protein levels, although the effects were milder in the case of proteins (Fig 7B).

EcR dsRNA treatment resulted in an increase in the fat body glycogen 36h PBM as revealed by means of PAS staining (Fig 7C). An increase in circulating sugars was observed in EcR-depleted female mosquitoes; in particular, the levels of glucose and fructose were highly elevated, reflecting an inability of these mosquitoes to utilize sugars (Fig 7D). Both glucose and fructose levels increased by greater than 3-fold at 36h PBM as result of EcR knockdowns. EcR dsRNA treatment resulted in an increase in TAG levels and a drop in the ATP levels 36h PBM (Fig 7E and 7F). To examine whether EcR promotes CM, PBM, via altering the glycolytic flux, we measured the IM levels downstream of glycolysis. Consistent with their inability to catabolize sugar reserves, EcR-silenced mosquitoes showed accumulation of early intermediates of the glycolytic pathway—glucose-6-phosphate and fructose-6-phosphate (Fig 7G). There was also a considerable build-up of lactate (Fig 7G). The level of pyruvate declined slightly, while that of citrate decreased considerably in these mosquitoes. These results clearly pointed to the fact that EcR is a critical regulator of CM during the PBM phase of the gonadotrophic cycle in female mosquitoes.

### Temporal coordination of the phosphoenolpyruvate carboxykinase (PEPCK)

PEPCK is an essential enzyme in maintaining glucose homeostasis and as such pays important role in response to stress and starvation [15,16,17]. The microarray and qPCR analysis has revealed that the level of the *PEPCK* gene transcript was high at the beginning of the PE phase, but it was dramatically reduced by 72h PE (Fig 1 and S7A Fig). In agreement with these data, the expression of the *PEPCK* gene was inhibited by the application of JH *in vivo* and activated by Met RNAi silencing, indicating negative regulation of this gene by the JH/Met in PE phase (S7B and S7C Fig). In contrast to most CM genes, activation of which reached maximum at 36h PBM, the *PEPCK* gene was highly upregulated immediately after a blood meal in *Aedes* females (Fig 1 and S7D Fig). Significantly, both *in vivo* and *in vitro* tissue culture experiments have shown that AAs play a key role in activating this gene expression (S7E and S7F Fig). However, these experiments have shown that 20E is not involved in regulation of this gene expression (S7E and S7F Fig). Furthermore, EcR RNAi silencing did not affect its transcript levels (S7G Fig).

### Temporal coordination of pentose phosphate pathway (PPP) genes during the gonadotrophic cycle

**Fig 8:**
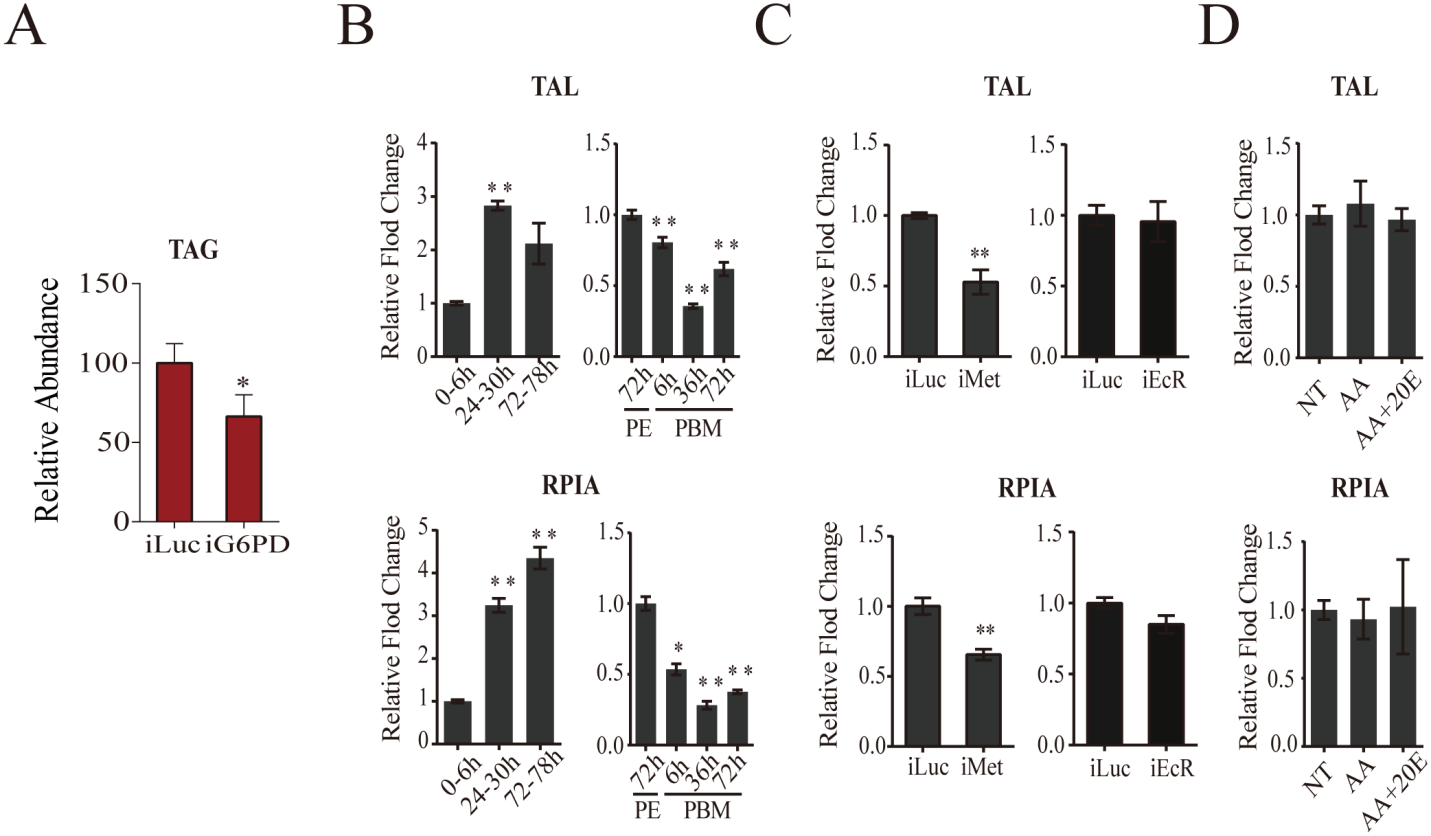
Analysis of pentose phosphate pathway genes. (A) A decrease in the TAG levels in *G6PD*-depleted adult female mosquitoes, as measured by colorimetry. (B) Expression of pentose phosphate pathway genes *TAL* (top) and *RPIA* (bottom) during PE and PBM. Sample points, sample collections and experimental procedures are similar to that of Fig 1C. (C) Effect of Met and EcR knockdowns on the expression of *TAL* (top) and *RPIA* (bottom) during PE and PBM. Sample collections and experiments are similar to that of Fig 4B (for iMet) and Fig 6A (for iEcR). (D) AA and 20E had no effect on *TAL* and *RPIA* expression. Sampling and experiments are similar to that of S5A Fig. The controls are represented as relative abundance of 100. Error bars represent ± SD. *p < 0.05;**p < 0.01.

The PPP consists of oxidative and non-oxidative branches [18]. In contrast to other CM pathways, the genes encoding PPP enzymes of both branches were transcriptionally active throughout the PE phase and downregulated during the PBM phase (Fig 1). In the PPP oxidative branch, glucose-6-phosphate is utilized for the synthesis of ribose-5-phosphate, with glucose-6-phosphate dehydrogenase being a rate-limiting enzyme [18]. In this respect, it was of particular interest that genes encoding glucose-6-phosphate dehydrogenase (G6PD) and ribose-5-phosphate isomerase A (RPIA) were sequentially activated during the PE phase (Fig 1). G6PD also reduces NADP+ to NADPH that is utilized in lipid biosynthesis [18]. In *Aedes* female mosquitoes, the expression of the gene encoding G6PD is regulated by Met (Fig 5A). G6PD RNAi depletion resulted in decreased TAG levels, suggesting that Met-dependent control of this enzyme contributes to fat metabolism in the mosquito fat body (Fig 8A).

Transketolase (TAL), which is the rate-limiting enzyme of the non-oxidative PPP branch [18], exhibited a higher expression level during the first 24h PE, while its expression was downregulated during the PBM phase (Fig 1). We used qPCR to examine the transcript abundance of genes encoding RPIA and TAL, representatives of the oxidative and non-oxidative PPP branches, respectively. This analysis confirmed the transcriptome data showing an elevation of transcript abundance of these two PPP genes at late PE phase and a decrease at 36h PBM (Fig 8B). RNAi depletion demonstrated that Met was an activator of expression of these genes, while EcR had no effect (Fig 8C). Moreover, *in vitro* fat body assay experiments confirmed the lack of 20E effect on *TAL* and *RPIA* expression (Fig 8D).

## Discussion

Throughout each gonadotrophic cycle, females of hematophagous mosquitoes undergo drastic physiological changes, shifting from nectar feeding and host seeking to blood utilization and rapid egg development. We show here that these changes are accompanied by CM reprogramming to support the dramatically different functional requirements of a reproducing female mosquito. To accommodate this reprogramming, the female mosquito fat body, which is the metabolic center, undergoes a particularly remarkable transformation. Our transcriptome and qPCR analyses have demonstrated that the expression of genes encoding CM enzymes in this tissue was synchronized with the two phases of the gonadotrophic cycle, responding to the varying energy requirements of the reproducing female mosquito. Protein levels of enzymes involved in glycolysis and glycogen/sugar metabolism exhibited periodic changes throughout the mosquito reproductive cycle that correlated with the transcript abundance of their respective genes. Levels of stored and circulating sugars revealed their periodic accumulation and depletion in response to changing energy requirements throughout. These sugar levels were concurrent with transcript levels of genes encoding glycogen/sugar metabolism enzymes. Metabolomics analysis provided further evidence that the CM dynamics were entirely different during the PE and PBM phases of the female mosquito gonadotrophic cycle. Moreover, these data corroborated with the existence of a link between levels of CM gene expression and IMs. In addition, our analysis has revealed that the timing and regulation of the PPP was different from other CM pathways, suggesting its pivotal role in metabolic homeostasis of the female mosquito. Overall, our analyses suggest that the temporal coordination of CM in female mosquitoes occurs to a large degree at the gene level.

We have demonstrated here that Met functions as a major regulatory switch that governs metabolic reprogramming during the PE phase of the female mosquito gonadotrophic cycle. Our data suggest that Met acts at the genomic level, affecting expression of CM genes, thus determining the PE CM reprogramming. The majority of genes involved in CM, including those of glycogen/sugar, glycolysis and the citrate cycle, were greatly elevated in Met-silenced female mosquitoes, while several genes encoding the PPP were downregulated. The JH receptor Met belongs to the bHLH-PAS family of heterodimeric transcription factors, proteins that respond to environmental or physiological signals and are involved in mediating multiple cell responses including metabolism and cancer [19,20]. The genomic action of Met has been established [21]—Met forms heterodimers with other bHLH-PAS factors in a JH-dependent manner and activates target genes via interaction with E-box motifs in their regulatory regions [22,23,24,25]. However, Met is also involved in the JH-mediated repression hierarchy. While the gene activation by Met appears to be direct, its repressive action requires intermediate factors [5]. In addition to a significant effect on CM enzymes at the transcript level, Met RNAi silencing caused elevation of glycolytic flux and depletion of stored and circulating sugars.

Ingestion of blood leads to dramatic events in a female mosquito. Our transcriptomics and metabolomics analyses have revealed that there is an immediate change in the CM status following blood feeding. The *TREA* transcript increase and the drop in the trehalose level at 6h PBM suggest an early onset of trehalose utilization for glycolysis. Likewise, there was a dramatic rise in the *LDH* transcript level as early as 3h PBM followed by a lactate spike. The citrate cycle IMs also exhibited early sharp increases at 6h PBM. This instant elevation of glycolysis to maintain high levels of glycolytic intermediates occurs prior to the rise of the 20E titer in the female mosquito, indicating that it is regulated by factors other than this hormone. Indeed, we found that the early PBM response is likely controlled by amino acids. In the *in vitro* fat body culture assay, the *LDH* gene transcript was elevated in response to amino acids, but downregulated by 20E. The role of the amino acid/TOR pathway in vitellogenic PBM events has been established [10]. The mosquito fat body serves as the nutrient sensor organ detecting signaling amino acids derived from a blood meal [1,26]. Here, we have uncovered the role of amino acids in regulating CM at the early PBM stage. *PEPCK* gene activation by AAs occurs at the onset of blood feeding, the time of the ingestion of a huge amount of food in a form of blood. This physiological state imposes an enormous energy requirement on a female mosquito that is needed for the rapid excretion of a large volume of fluid and digestion of a massive blood meal. It appears that a high elevation of the *PEPCK* gene expression that correlates with these events is essential for maintaining circulating sugar homeostasis and represents an adaptation of mosquito CM to hematophagy.

The PBM stage is the apex of the gonadotrophic cycle, when a female mosquito utilizes a huge blood meal and rapidly develops over a hundred eggs just within 48h. We show here that there is a stunningly high level of the CM activity during the middle of the PBM stage, particularly of glycolysis. The genes encoding the rate-limiting glycogen and glycolytic enzymes, such as *GLY*, *PFK* and *PYK*, were upregulated 40 to over 100-fold by 36h PBM. Apart from providing substrate for energy production, the major function of aerobic glycolysis is to maintain high levels of glycolytic intermediates to support anabolic reactions in rapidly dividing cells [6]. Our analysis of CM IMs showed that the glycolytic flux was extremely elevated at the PBM stage.

20E is the major hormone controlling events of the PBM stage of the female mosquito reproductive cycle, and its action is mediated by the heterodimer of EcR and the insect RXR homologue Ultraspiracle, both of which are members of the nuclear receptor superfamily [4]. Nuclear receptors are a specialized family of ligand-bound or unliganded transcription factors that play central roles in regulating development, growth and metabolism [27]. In female mosquitoes, the 20E regulatory hierarchy is responsible for the *YPP* gene expression in the fat body [28,29,30]. Our results further suggest that control of CM occurs mainly at the gene level, and EcR is an important regulator of these genes during dramatic increases in CM. In rapidly developing *Drosophila melanogaster* larvae, CM is temporally coordinated by the estrogen-related receptor (ERR) [8]. This nuclear receptor alters the expression of genes encoding metabolic pathway enzymes, thus playing the role of a metabolic switch. Whether ERR plays a similar role in the female mosquito and its mode of interaction with EcR in synchronizing CM during the PBM stage requires further study.

In summary, we have presented a comprehensive analysis of CM dynamics in the female mosquito during the reproductive cycle. We show that such metabolism is tightly correlated with the rapidly changing physiological conditions of this organism. Our transcriptomics and metabolomics studies have revealed the association of expression of genes encoding CM pathways and IMs. Our analyses have identified that Met is the key regulatory switch responsible for temporal coordination of CM during the PE phase of the female mosquito gonadotrophic cycle. We also show that 20E/EcR and amino acids play different roles in CM regulation. Further molecular analysis of these metabolic regulatory pathways may lead to the implementation of metabolism-based methods to prevent mosquito-borne disease transmission.

## Materials and Methods

### Experimental animals

The mosquito *A*. *aegypti* Rockefeller strain was raised as described previously [11,31]. Adult mosquitoes were fed water and 10% sucrose solution continuously. All procedures for vertebrate animal use were approved by the Institute of Zoology Animal Care and Use Committee.

### Metabolic profiling

Sample sets from 12 independent mosquito populations were analyzed for every experimental condition. Six mosquitoes per sample point were washed in PBS buffer, frozen in liquid nitrogen, grounded in 400 μl pre-cooled 90% MeOH and then incubated for 1 h at -20°C [32]. Following centrifugation and debris removal, a second extraction step with 60% MeOH was performed. The supernatant was vacuum dried for 1 h and incubated with 40 μl O-methoxylamine hydrochloride (20 mg/ml saturated in pyridine) for 1 h at 37°C. Then, 50 μl MSTFA reagent was added to the samples, which were then incubated for 30 min at 37°C, with shaking, and finally diluted with 400 μl n-hexane and transferred to the auto sampler vials for the next step. GC-MS analysis was performed following a standard protocol using Agilent 7890 GC coupled with a 5975N series mass selective detector (MSD). The following temperature steps were used: initial temperature of 75°C for 1 min, 5°C /min ramp to 250°C for 5 min, 5°C/min ramp to 320°C for 3 min. A 1-μl sample was injected in split-less mode at 250°C with helium carrier gas flow set at 1 ml/min. A HP-5MS column with a 5-m-long guard column was used for the analysis. Chromatogram acquisition, peak de-convolution and library searches were performed using Agilent MSD Chemstation software. Metabolites were identified using authentic chemical standards analyzed on the same system.

### Glycogen, TAG, and ATP measurements

Glycogen assays were performed as described previously [33,34]. SpectraMax Plus384 was used for detection. Six independent biological samples, with six adult female mosquitoes per sample, were used for each experimental condition. Following centrifugation, samples were transferred into 96-well plates, incubated with Free Glycerol Reagent (Sigma), and assayed using SpectraMax Plus384. For TAG measurements, six mosquitoes were homogenized in 100 μl PBST containing 0.5% Tween-20 and incubated at 70°C for 5 min. Then, the samples were incubated with Triglyceride Reagent (Sigma) and assayed colorimetrically. For ATP measurements, six mosquitoes were homogenized in extraction buffer (6 M guanidine-HCL, 100 mM Tris, 4 mM EDTA) and boiled for 5 min. After centrifugation, the supernatant was filtered via PTFE membrane for HPLC assays performed using an Agilent 1100 HPLC coupled with DVD detector, following a published protocol [35]. On the chromatogram, ATP peaks were identified by utilizing retention time of standards (Molecular Probes, 911734). Total glycogen, TAG, and ATP concentrations were normalized to endogenous protein level of the samples, determined using Bradford assays (BioRad, 500–0201).

### Glycogen staining

For histochemical analysis of fat body glycogen content, staining and visualization were performed as previously described [36]. The abdomen was separated from the rest of the body and fixed in 4% paraformaldehyde at 4°C overnight. Each sample was then dehydrated with increasing concentrations of ethanol, embedded in paraffin, and sectioned into 3- to 5-μm slices. Abdominal fragments were stained according to the periodic acid Schiff (PAS) method (Sigma, 395B) and observed under a Nikon Ni-E microscope.

### Micro-injection of dsRNA and application of hormones

dsRNA synthesis was performed as previously described [5]. The bacterial luciferase gene was used to generate control iLuc dsRNA. A Nanoliter 2000 injector (World Precision Instrument) was used to introduce corresponding dsRNA into the thorax of cold-anesthetized mosquito females 24h PE. The specificity of gene knockdown was characterized by a 50–70% decrease in transcript abundance of target genes (S3B and S4A Figs). All primers used for making dsRNA are listed in S5 Table. To test the effect of JH, 0.5 μl of JH (10 μg/ml JH in acetone as solvent) or acetone was topically applied to newly eclosed female mosquito abdomens. The females were examined 20h post treatment as previously described [24]. For metabolite measurements, samples were collected 20h post JH treatment. To test the effect of 20E, 0.5 μl 10^−6^ M 20E was injected along with amino acids into 72h PE female mosquitoes. Mosquitoes were examined 20h post treatment. Experiments were performed in triplicates under the same condition.

### RNA preparation and qPCR analysis

Total RNA samples were prepared under three different conditions, and fat bodies were dissected from abdomens of 10–15 individual mosquitoes. qPCR reaction was performed on the MX3000P system (Stratagene, CA) using SYBR green PCR Master Mix (Tiangen, Beijing). Thermal cycling conditions were: 94°C, 5 s; 59°C, 20 s; and 72°C, 20 s. Quantitative measurements were performed in triplicate and normalized to the internal control S7 ribosomal protein mRNA for each sample. Primers used for qPCR are listed in S5 Table.

### Western blot analysis

Eight mosquito fat bodies were homogenized in 100 μl of breaking buffer by pellet pestle, as described previously [37]. Aliquots of whole mosquito protein samples were resolved on 4–15% gradient SDS-polyacrylamide gels (Bio-Rad) and transferred to PVDF membranes (Invitrogen). After blocking, the membranes were incubated overnight with the primary antibody at 4°C (S3 Table). As loading control, an antibody against β-actin (Sigma) was used.

### Bioinformatics and statistical analysis

DEG datasets from PE and PBM time course microarray were utilized to reconstruct the expression profiles of the genes involved in metabolism. Complete linkage hierarchical clustering was performed using the hclust function in R [5]. Discrete clusters were obtained by cutting the resulting dendrogram with the cutree function using a visually determined height value. Orthologous groups and pathway information, based on the Kyoto Encyclopedia of Genes and Genomes (KEGG), were downloaded from the database [38] and used in this study. An enrichment analysis was used to detect the significance of alteration of each metabolic pathway, and *p* values were calculated based on hyper-geometric tests, as described previously [39]. In all other experiments, statistical significance was defined by a *p* value < 0.01, as evaluated using paired-end, two-tailed, student's *t*-tests (Graphpad 5.0). Comparisons were made between time points/ treatments and the controls and significant differences were indicated in the graphs. All quantitative data are reported as mean ± SD.

### *In-vitro* fat body culture

*In vitro* fat body culture experiments were performed as previously described [11,26]. Female mosquito abdominal walls with adhered fat body tissue were incubated in a culture medium under various conditions. In the culture medium lacking amino acids, an equal molar amount of mannitol was supplemented to compensate for changes in osmotic pressure [26]. 20E was added to the culture medium supplemented with a complete set of amino acids [26]. To mimic a natural rise in the 20E titer, the tissue was first incubated with 5 x 10^−8^ M of this hormone for 4h and then in the presence of 10^−6^ M for 4 h. Total RNA was then isolated and transcript abundance was analyzed using qPCR. The experiment was repeated three times under the same conditions.

## Supporting Information

S1 Fig CM gene expression dynamics in the fat body of adult female mosquitoes. (A) A schematic diagram showing genes encoding pathway enzymes for glycogen/sugar metabolism and glycolysis. Genes that based on the microarray data exhibited a greater than four fold down-regulation at 72h PE and up-regulation at 36h PBM are marked in pink. (B and C) KEGG based analysis of CM pathway gene cohorts in PE and PBM. Each bar represents a total number of genes of a given CM pathway in the *Ae*. *aegypti* genome, while the number of genes that are significantly enriched in a given time is marked by a darker tone. (B) Gene categories enriched at 6h PE (red) and 24h PE (blue). (C) Gene categories enriched at 12h PBM (red), 24h PBM (blue) and 36h PBM (green). Genes P values show the enrichment of genes in each respective pathway.
(TIF)

S2 Fig qPCR based transcript levels of additional genes encoding enzymes of the CM pathway during the reproductive development in adult female mosquitoes *Ae*. *aegypti*. Transcripts of PE time points were normalized to the level of 0-6h PE, while that of PBM were normalized to that of 72h PE. Error bars represent ± SD. *p < 0.05;**p < 0.01.
(TIF)

S3 Fig Effects of JH on CM genes and metabolites. (A) qPCR analysis of selected CM genes in the female mosquitoes after topical application of JH III. Tissues were isolated 20h post treatment and subjected to qPCR analysis. (B) The level of glycogen, glucose and TAG in female mosquitoes after the same treatment. All experiments were performed in triplicate, with similar results. Error bars represent ± SD. * *p* < 0.05;** *p* < 0.01.
(TIF)

S4 Fig **Effect of JH receptor Met on CM during PE** (A) KEGG based enrichment analysis for CM pathway genes in Met depleted mosquito transcriptome. The iMet upregulated transcriptome is significantly enriched in glycolysis and glycogen/sugar metabolism genes. The number of iMet upregulated transcripts belonging to a particular pathway is marked in darker tones. The bars represent total number of mosquito genes in each CM pathway analyzed. (B) The level of knockdown of Met transcripts in dsMet-injected adult female mosquitoes. (C) Expression of CM genes in Met-depleted mosquito in comparison to iLuc controls. Fat body RNA was collected 5 days post injection and analyzed by qPCR. Error bars represent ± SD. *p < 0.05;**p < 0.01.
(TIF)

S5 Fig Differential effect of AAs and 20E on expression of selected CM genes. (A). Effect of AA and 20E on CM gene expression in *in vitro* fat body culture. NT, culture medium without AA and 20E; AA—culture medium supplemented with AAs; AA+20E, culture medium supplemented with AAs and 20E. Fat bodies dissected from 72h PE adult female mosquitoes were incubated in culture medium with AA or AA+20E for 8hrs. Fat bodies cultured on minimal media were used as controls (NT). The tissue was harvested for RNA isolation and qPCR analysis. (B) Effect of AA and 20E *in vivo*. Female mosquitoes 72h PE were injected with 20E, AAs or a combination of 20E and AAs. Injection with ethanol (solvent) served as a control. Tissues were isolated 20h post injections and subjected to qPCR analysis. (C) A decrease of glycogen and glucose levels was observed in female mosquitoes treated with 20E and AAs. Error bars represent ± SD. * *p* < 0.05, ** *p* < 0.01.
(TIF)

S6 Fig The effect of EcR RNAi silencing. (A) A decrease of laid egg numbers was observed in EcR-depleted adult female mosquitoes. (B) Effective knockdown of EcR transcripts in dsEcR injected adult female mosquitoes. (C) Expression of additional CM genes in EcR-depleted mosquitoes in comparison to iLuc controls. The mosquitoes were blood fed five days after injection with dsEcR, the fat body RNA was collected 36h PBM and subjected to qPCR analysis. Error bars represent ± SD. *p < 0.05;**p < 0.01.
(TIF)

S7 Fig Temporal coordination of the phosphoenolpyruvate carboxykinase (PEPCK). (A) qPCR analysis of the *PEPCK* gene transcript abundance during PE. Relative abundance of PE time points were normalized to the 0-6h PE. In the graphs, the abundance of these two time points is represented as 1.0, with corresponding adjustments for other time points. (B) Effect of Met and EcR knockdowns on the expression of *PEPCK* during PE and PBM. Sample collections and experiments are similar to that of Fig 4B (for iMet) and Fig 6A (for iEcR). (C) Expression of the *PEPCK* gene in Met-depleted mosquitoes in comparison to that in iLuc control. (D) qPCR analysis of the *PEPCK* gene transcript abundance during PBM. Relative abundance of PBM time points were normalized to the 72h PE. In the graphs, the abundance of these two time points is represented as 1.0, with corresponding adjustments for other time points. (E) Effect of AAs and 20E on CM gene expression. A clear induction of the *PEPCK* gene by AAs was observed in in-vitro fat body culture experiments. Addition of 20E (AA +20E) did not result in further induction of this gene. The experiments were performed as in S4 Fig. Fat bodies dissected from 72h PE adult female mosquito were incubated in culture medium with AA or AA+20E for 8 hrs. Fat bodies cultured on minimal media were used as controls (NT). The tissue was harvested for RNA isolation and qPCR analysis. (F) Effect of AA and 20E *in vivo*. Female mosquitoes 72h PE were injected with 20E, AAs or a combination of 20E and AAs. Injection with ethanol (solvent) served as a control. Tissues were isolated 20h post injections and subjected to qPCR analysis. (G) Expression of the *PEPCK* gene in EcR-depleted mosquitoes in comparison to iLuc controls. The mosquitoes were blood fed five days after injection with dsEcR, the fat body RNA was collected 36h PBM and subjected to qPCR analysis. All experiments were performed in triplicate, with similar results. Error bars represent ± SD. ** *p* < 0.01.
(TIF)

S1 Table Temporal Expression of CM genes during PE development. Genes encoding pathway enzymes of glycogen/sugar metabolism, glycolysis, citrate cycle and pentose phosphate pathway are provided. Differential expression was calculated using a minimum fold change of ≥1.75 (0.8 in a log2 scale) as the confidence threshold and a false-discovery rate (*P* value) of ≤0.01 (Zou et al., 2013).
(XLSX)

S2 Table Temporal Expression of CM genes during PBM development. Genes encoding pathway enzymes of glycogen/sugar metabolism, glycolysis, citrate cycle and pentose phosphate pathway are provided. Differential expression of during PBM was calculated by comparing transcripts from each of the nine-time point with that of 72h PE using the same filtering criteria as those for the PE genes.
(XLSX)

S3 Table List of antibodies used for western blot analysis. Owing to the conservation of CM pathway and pathway enzymes, commercially antibodies against CM enzymes from other species can be utilized to detect *Aedes* proteins.
(XLSX)

S4 Table CM gene expression in Met depleted mosquito. Differential gene expression in iLuc and iMet was calculated by comparison with 72h PE mosquito with a false discovery rate (*P* value) ≤0.01 (Zou et al., 2013). The control 72h PE was compared to 6h PE.
(XLSX)

S5 Table Primers used for qPCR and RNAi. (XLSX)
