# Supplementary figures and images for: Temporal Coordination of Carbohydrate Metabolism during Mosquito Reproduction
Source: PLoS Genet. 2015 Jul 9;11(7):e1005309. doi: 10.1371/journal.pgen.1005309 (PMC4497655; doi:10.1371/journal.pgen.1005309)

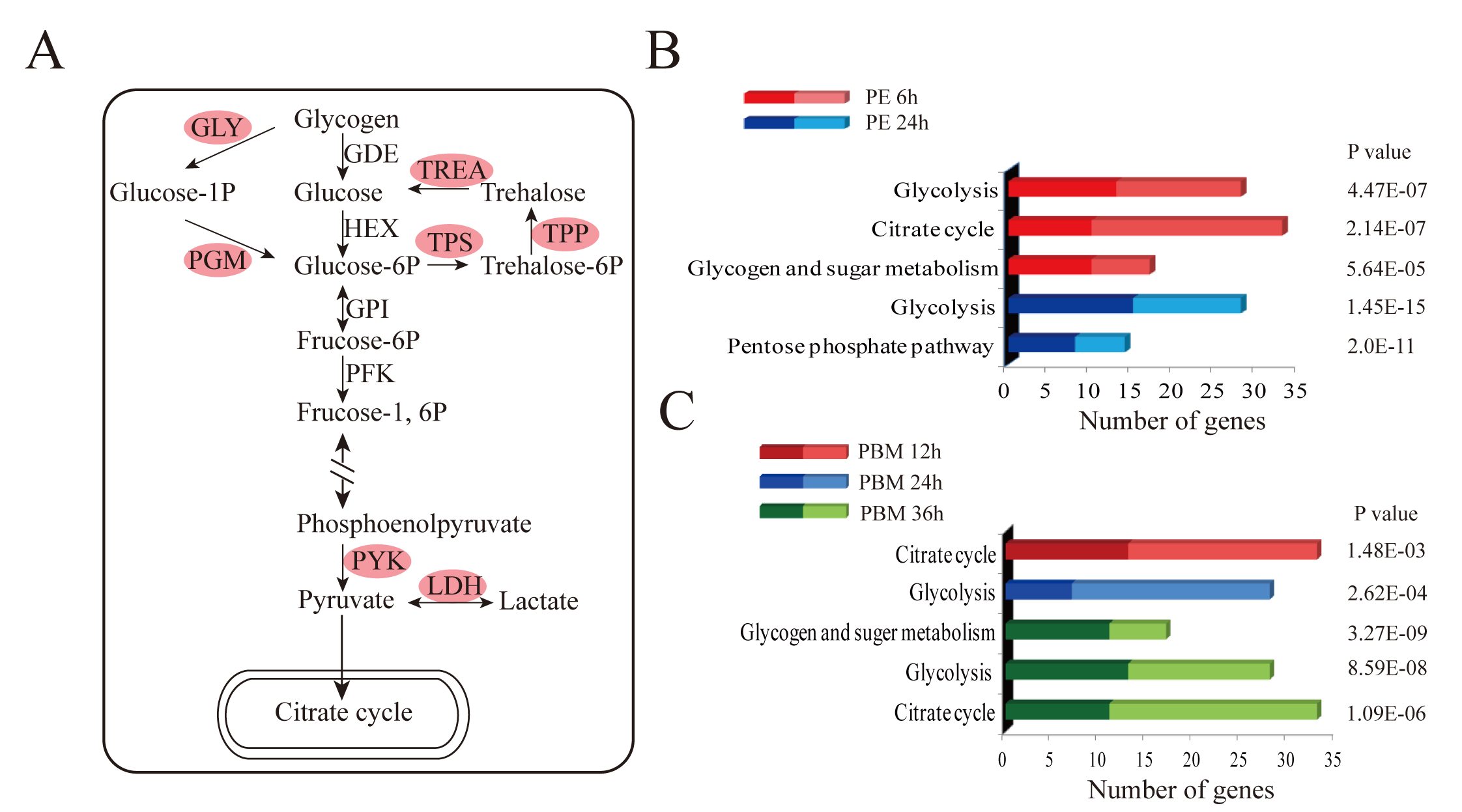

Supplement: S1 Fig — (A) A schematic diagram showing genes encoding pathway enzymes for glycogen/sugar metabolism and glycolysis. Genes that based on the microarray data exhibited a greater than four fold down-regulation at 72h PE and up-regulation at 36h PBM are marked in pink. (B and C) KEGG based analysis of CM pathway gene cohorts in PE and PBM. Each bar represents a total number of genes of a given CM pathway in the Ae. aegypti genome, while the number of genes that are significantly enriched in a given time is marked by a darker tone. (B) Gene categories enriched at 6h PE (red) and 24h PE (blue). (C) Gene categories enriched at 12h PBM (red), 24h PBM (blue) and 36h PBM (green). Genes P values show the enrichment of genes in each respective pathway. (TIF) [file pgen.1005309.s001.tif]

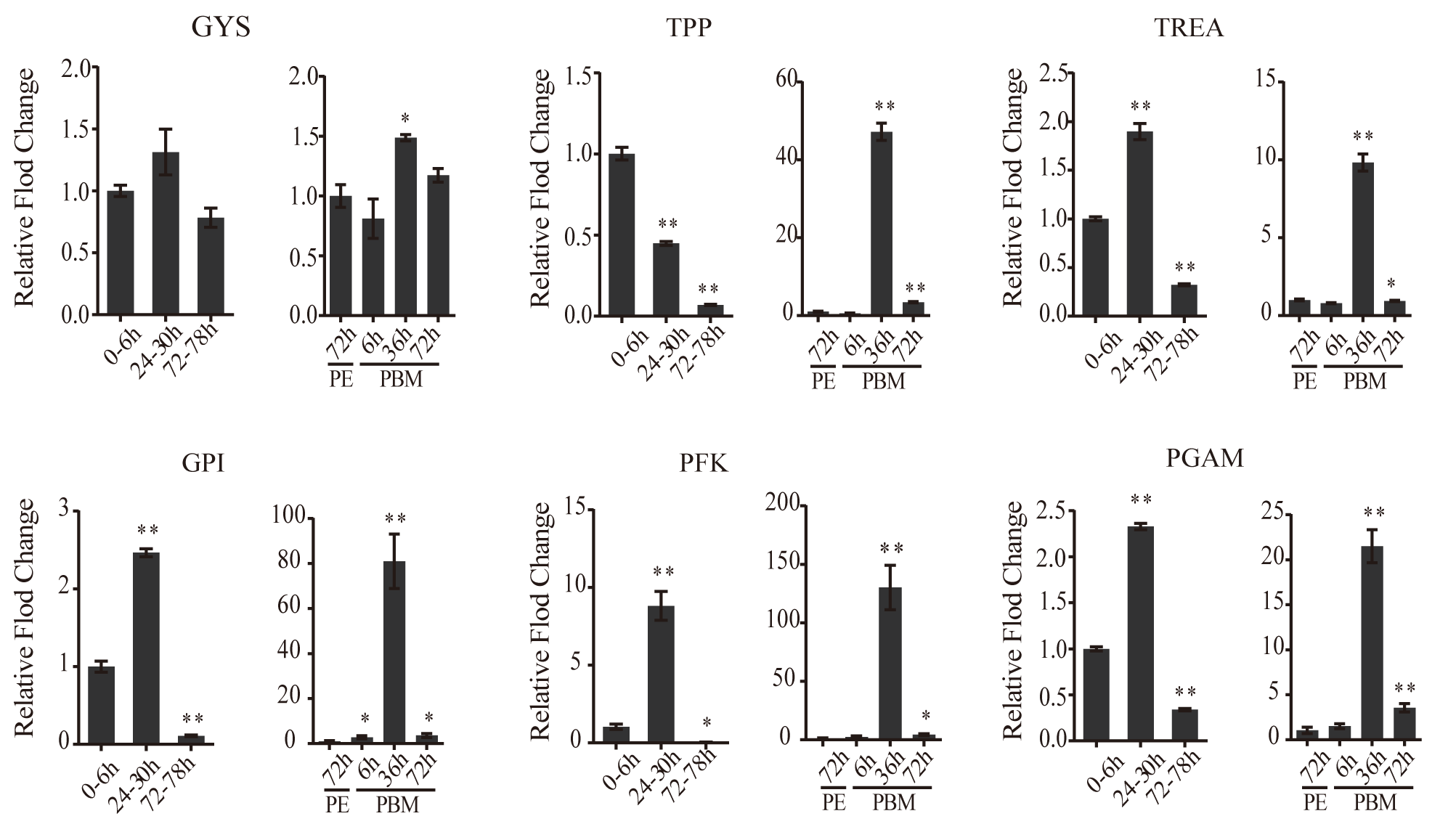

Supplement: S2 Fig — Transcripts of PE time points were normalized to the level of 0-6h PE, while that of PBM were normalized to that of 72h PE. Error bars represent ± SD. *p < 0.05;**p < 0.01. (TIF) [file pgen.1005309.s002.tif]

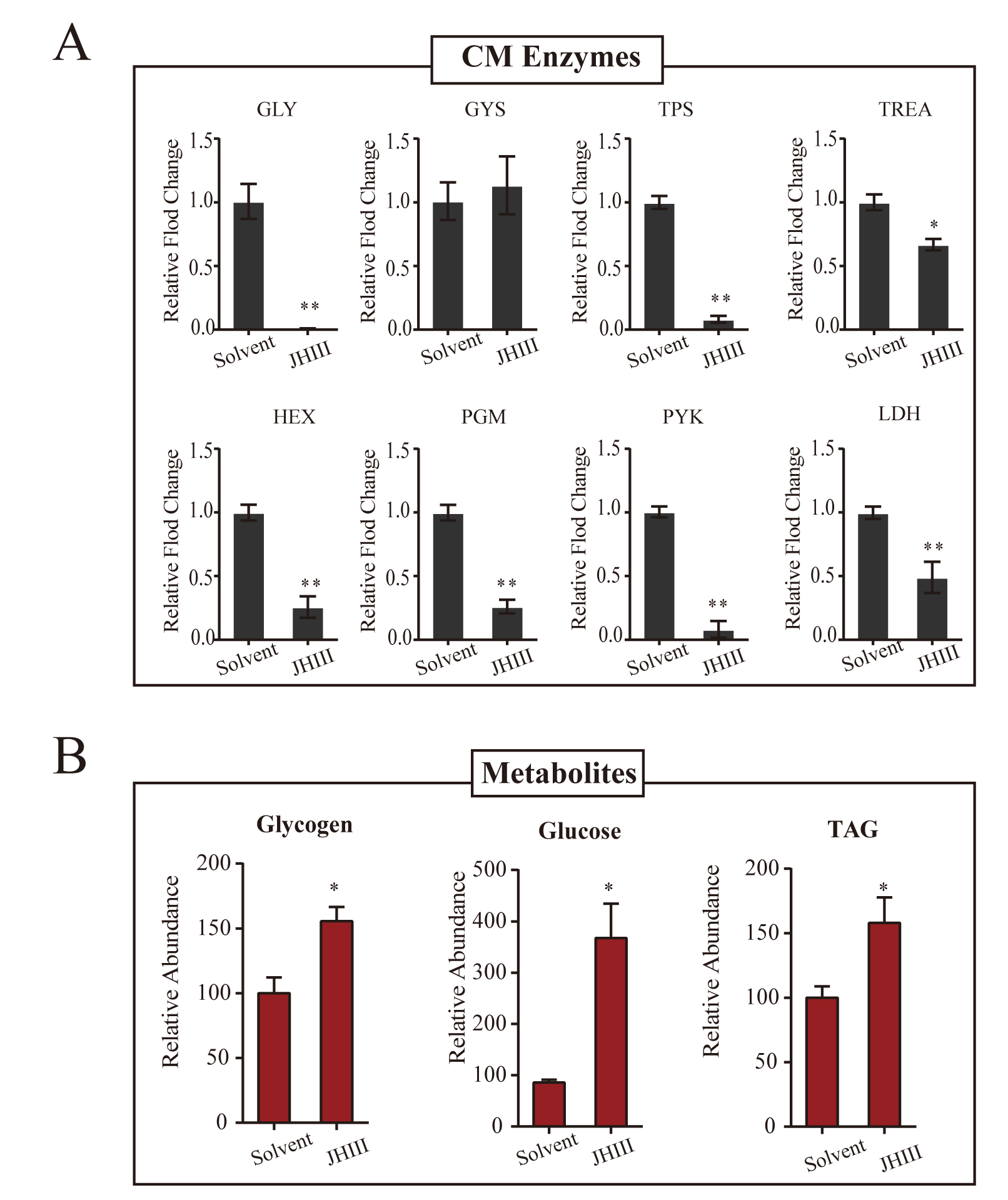

Supplement: S3 Fig — (A) qPCR analysis of selected CM genes in the female mosquitoes after topical application of JH III. Tissues were isolated 20h post treatment and subjected to qPCR analysis. (B) The level of glycogen, glucose and TAG in female mosquitoes after the same treatment. All experiments were performed in triplicate, with similar results. Error bars represent ± SD. * p < 0.05;** p < 0.01. (TIF) [file pgen.1005309.s003.tif]

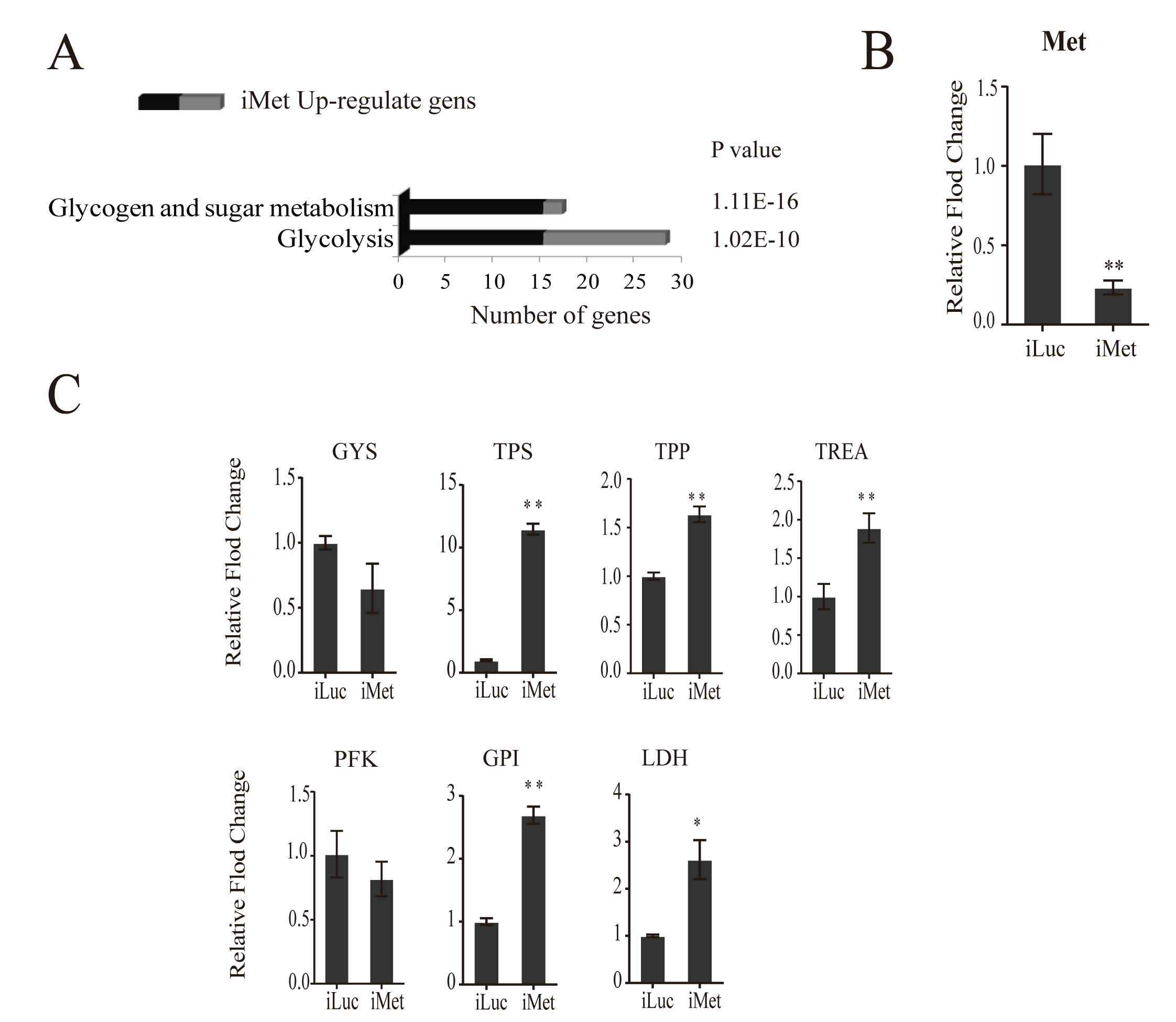

Supplement: S4 Fig — Effect of JH receptor Met on CM during PE (A) KEGG based enrichment analysis for CM pathway genes in Met depleted mosquito transcriptome. The iMet upregulated transcriptome is significantly enriched in glycolysis and glycogen/sugar metabolism genes. The number of iMet upregulated transcripts belonging to a particular pathway is marked in darker tones. The bars represent total number of mosquito genes in each CM pathway analyzed. (B) The level of knockdown of Met transcripts in dsMet-injected adult female mosquitoes. (C) Expression of CM genes in Met-depleted mosquito in comparison to iLuc controls. Fat body RNA was collected 5 days post injection and analyzed by qPCR. Error bars represent ± SD. *p < 0.05;**p < 0.01. (TIF) [file pgen.1005309.s004.tif]

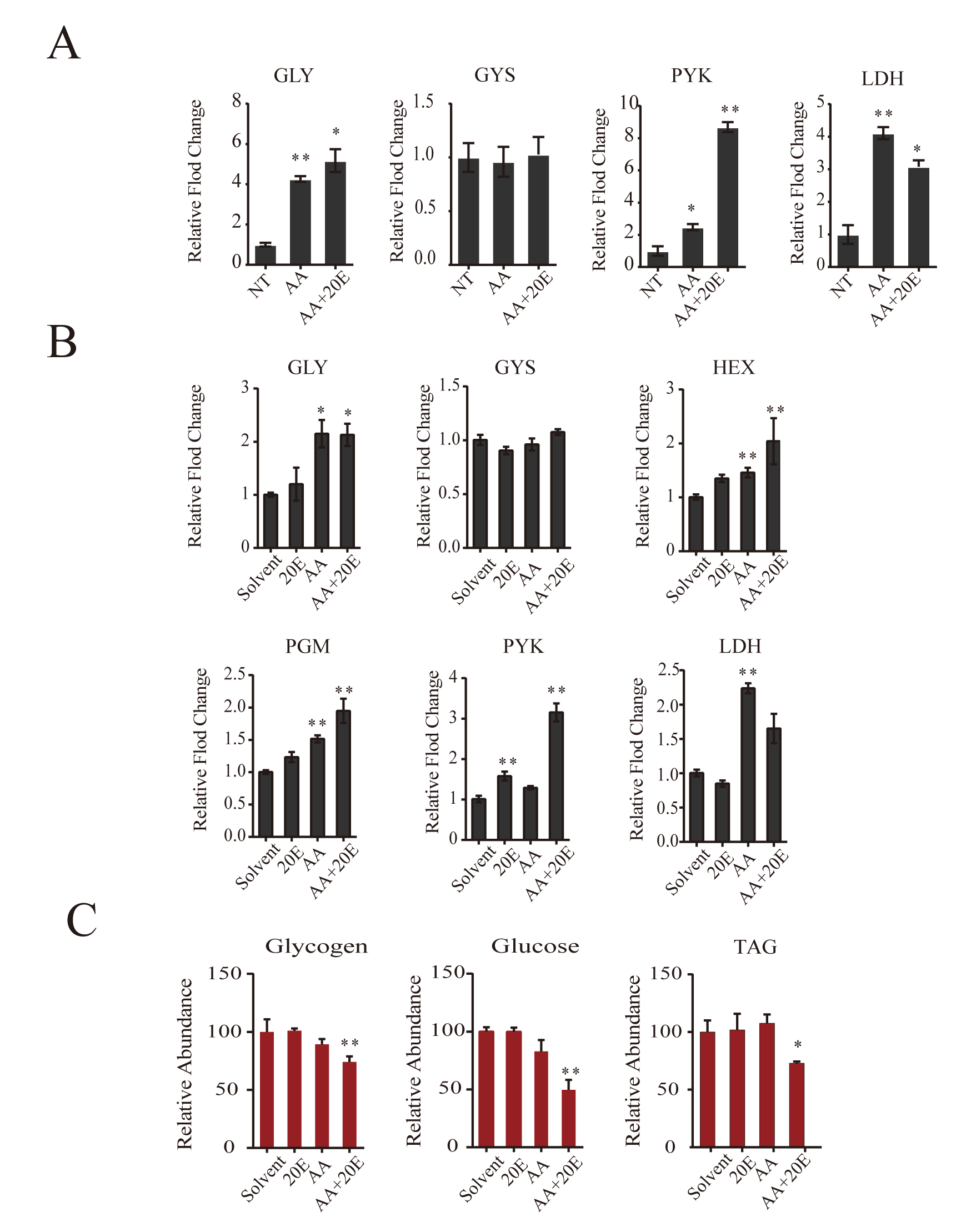

Supplement: S5 Fig — (A). Effect of AA and 20E on CM gene expression in in vitro fat body culture. NT, culture medium without AA and 20E; AA—culture medium supplemented with AAs; AA+20E, culture medium supplemented with AAs and 20E. Fat bodies dissected from 72h PE adult female mosquitoes were incubated in culture medium with AA or AA+20E for 8hrs. Fat bodies cultured on minimal media were used as controls (NT). The tissue was harvested for RNA isolation and qPCR analysis. (B) Effect of AA and 20E in vivo. Female mosquitoes 72h PE were injected with 20E, AAs or a combination of 20E and AAs. Injection with ethanol (solvent) served as a control. Tissues were isolated 20h post injections and subjected to qPCR analysis. (C) A decrease of glycogen and glucose levels was observed in female mosquitoes treated with 20E and AAs. Error bars represent ± SD. * p < 0.05, ** p < 0.01. (TIF) [file pgen.1005309.s005.tif]

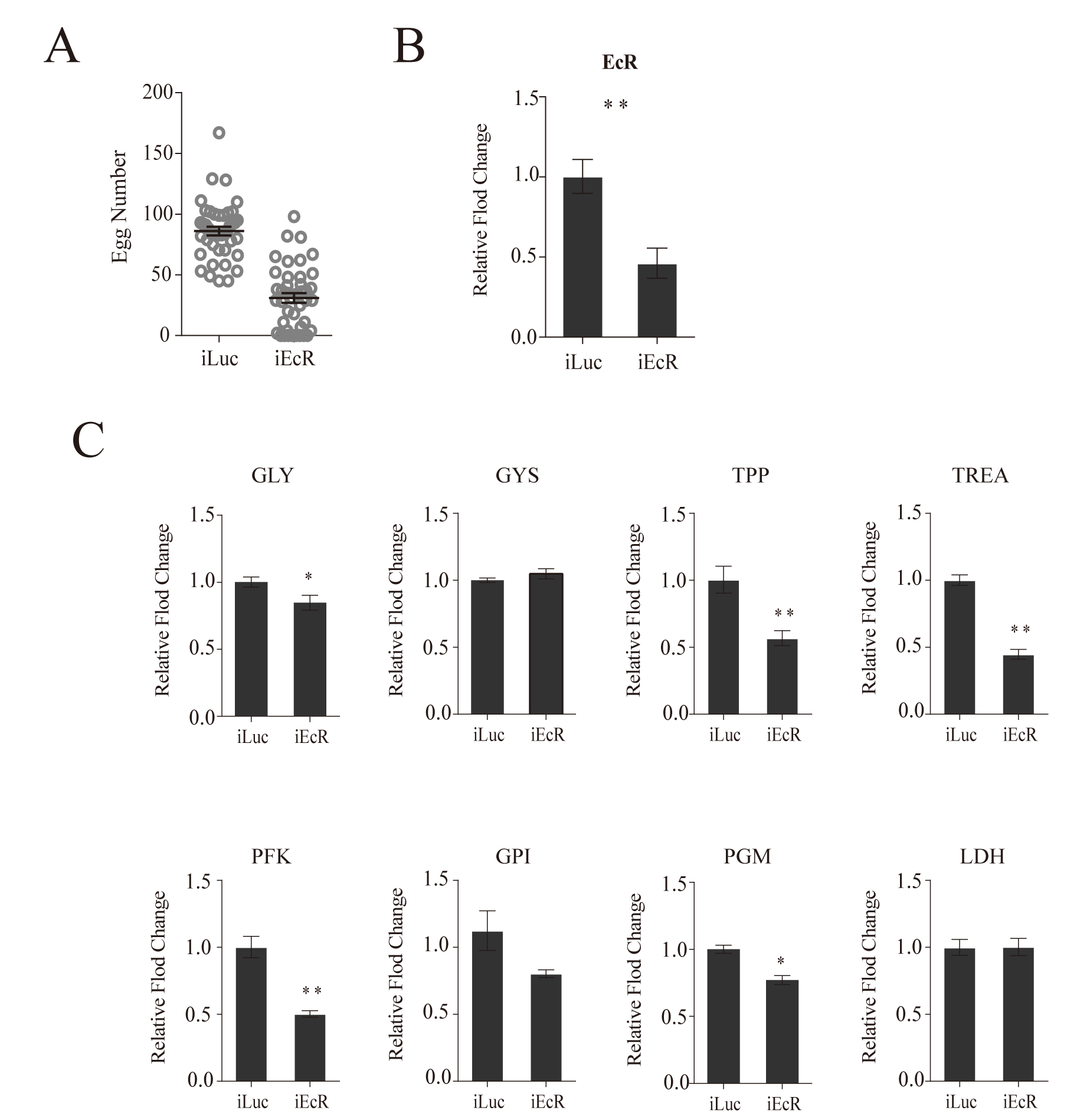

Supplement: S6 Fig — (A) A decrease of laid egg numbers was observed in EcR-depleted adult female mosquitoes. (B) Effective knockdown of EcR transcripts in dsEcR injected adult female mosquitoes. (C) Expression of additional CM genes in EcR-depleted mosquitoes in comparison to iLuc controls. The mosquitoes were blood fed five days after injection with dsEcR, the fat body RNA was collected 36h PBM and subjected to qPCR analysis. Error bars represent ± SD. *p < 0.05;**p < 0.01. (TIF) [file pgen.1005309.s006.tif]

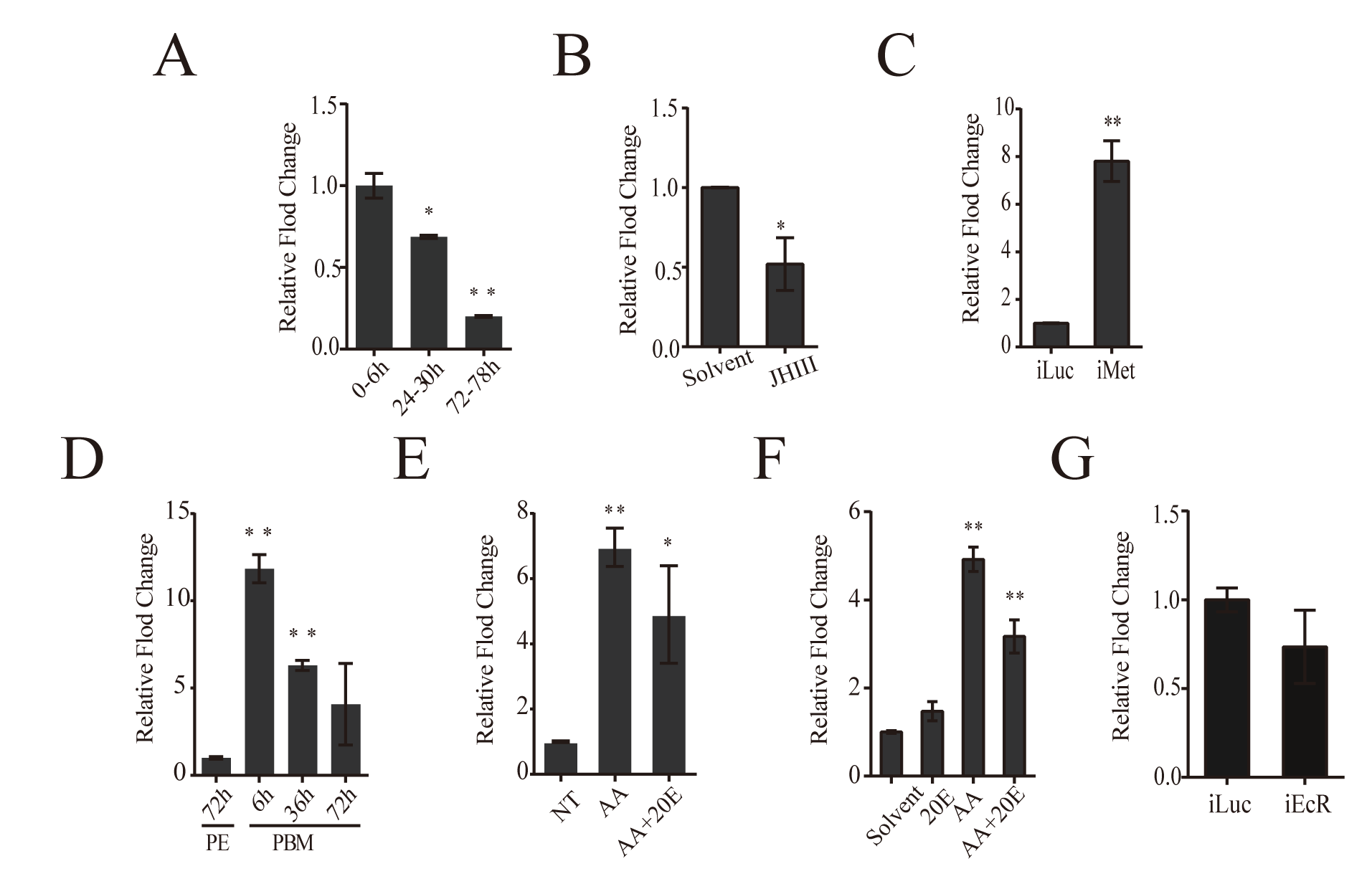

Supplement: S7 Fig — (A) qPCR analysis of the PEPCK gene transcript abundance during PE. Relative abundance of PE time points were normalized to the 0-6h PE. In the graphs, the abundance of these two time points is represented as 1.0, with corresponding adjustments for other time points. (B) Effect of Met and EcR knockdowns on the expression of PEPCK during PE and PBM. Sample collections and experiments are similar to that of Fig 4B (for iMet) and Fig 6A (for iEcR). (C) Expression of the PEPCK gene in Met-depleted mosquitoes in comparison to that in iLuc control. (D) qPCR analysis of the PEPCK gene transcript abundance during PBM. Relative abundance of PBM time points were normalized to the 72h PE. In the graphs, the abundance of these two time points is represented as 1.0, with corresponding adjustments for other time points. (E) Effect of AAs and 20E on CM gene expression. A clear induction of the PEPCK gene by AAs was observed in in-vitro fat body culture experiments. Addition of 20E (AA +20E) did not result in further induction of this gene. The experiments were performed as in S4 Fig. Fat bodies dissected from 72h PE adult female mosquito were incubated in culture medium with AA or AA+20E for 8 hrs. Fat bodies cultured on minimal media were used as controls (NT). The tissue was harvested for RNA isolation and qPCR analysis. (F) Effect of AA and 20E in vivo. Female mosquitoes 72h PE were injected with 20E, AAs or a combination of 20E and AAs. Injection with ethanol (solvent) served as a control. Tissues were isolated 20h post injections and subjected to qPCR analysis. (G) Expression of the PEPCK gene in EcR-depleted mosquitoes in comparison to iLuc controls. The mosquitoes were blood fed five days after injection with dsEcR, the fat body RNA was collected 36h PBM and subjected to qPCR analysis. All experiments were performed in triplicate, with similar results. Error bars represent ± SD. ** p < 0.01. (TIF) [file pgen.1005309.s007.tif]
